# Supplementary material for: Munc13-1 restoration mitigates presynaptic pathology in spinal muscular atrophy
Source: Nat Commun. 2025 Sep 30;16:8724. doi: 10.1038/s41467-025-64164-w (PMC12485113; doi:10.1038/s41467-025-64164-w)
Supplement: Supplementary file 1 — Supplementary Information [file 41467_2025_64164_MOESM1_ESM.pdf]

Supplementary Information for

Munc13-1 Restoration Mitigates Presynaptic Pathology in Spinal Muscular Atrophy

Mehri Moradi, Julia Weingart, Chunchu Deng, Mahoor Nasouti, Michael Briese, Sibylle Jablonka, Markus Sauer, Michael Sendtner

This PDF file includes:

Supplementary Figures 1 to 8 and Supplementary Table 1

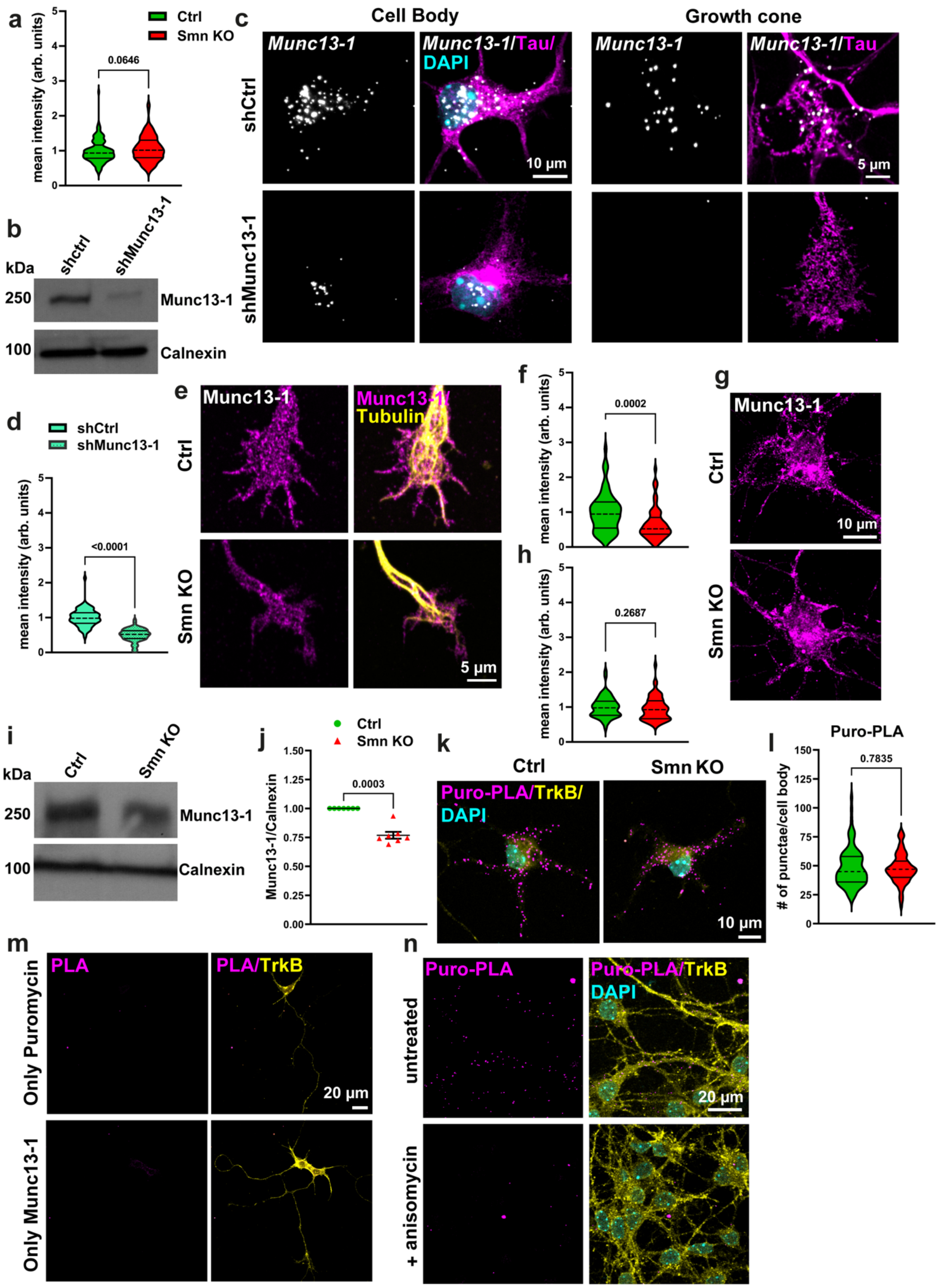

Supplementary Fig. 1 Munc13-1 expression is not altered in somata of Smn KO motoneurons. (**a**) Quantification of smFISH indicates similar somatic Munc13-1 mRNA levels in cultured Smn KO and control motoneurons (Ctrl) (n = 140-186 cells from n = 2 biological

replicates). (**b**) Immunoblot of total lysates from cultured motoneurons transduced with Munc13-1-targeting shRNA or control shRNA lentiviruses shows reduced Munc13-1 levels following knockdown. Calnexin served as loading control (representative of n = 2 biological replicates). (**c**) Representative images of somata and axonal growth cones of cultured control and Munc13-1 knockdown motoneurons showing Munc13-1 FISH signal. (**d**) Munc13-1 FISH signal is reduced in somata of Munc13-1 knockdown motoneurons (****P < 0.0001; n = 60-76 cells from n = 2 biological replicates). (**e**) Representative images of axonal growth cones of cultured motoneurons stained against Munc13-1 and Tubulin. (**f**) Graph reveals decreased Munc13-1 immunoreactivity in axonal growth cones of cultured Smn KO motoneurons (***P = 0.0002; n = 61-62 cells from n = 3 biological replicates). (**g**) Immunostaining of Munc13-1 in somata of cultured motoneurons. (**h**) Quantification shows unaltered Munc13-1 protein levels in somata of cultured Smn KO motoneurons (n = 62-63 cells from n = 3 biological replicates). (**i**) Immunoblot analysis of total lysates from cultured Smn KO and control motoneurons showing Munc13-1 total protein levels. Calnexin served as loading control. (**j**) Quantification of immunoblots reveals 25% reduction in Munc13-1 total protein levels in Smn KO compared to control (***P = 0.0003; n = 7 biological replicates). (**k**) Representative images of somata from cultured motoneurons showing Munc13-1-puromycin-Proximity Ligation Assay (Munc13-1-Puro-PLA) signal. (**l**) Graph shows similar somatic Munc13-1-Puro-PLA signal in Smn KO and control motoneurons (n = 87-95 cells from n = 3 biological replicates). (**m**) No PLA signal is detectable in wt motoneurons when only Munc13-1 or puromycin antibodies are incubated. (**n**) Munc13-1-Puro-PLA signal is almost completely abolished following pretreatment of motoneurons with anisomycin. Two-tailed Mann-Whitney U test in **a**, **d**, **f**, **h**, and **l**, and One-tailed Mann-Whitney U test in **j**. Bars represent mean ± SEM. Source data are provided as a Source Data file.

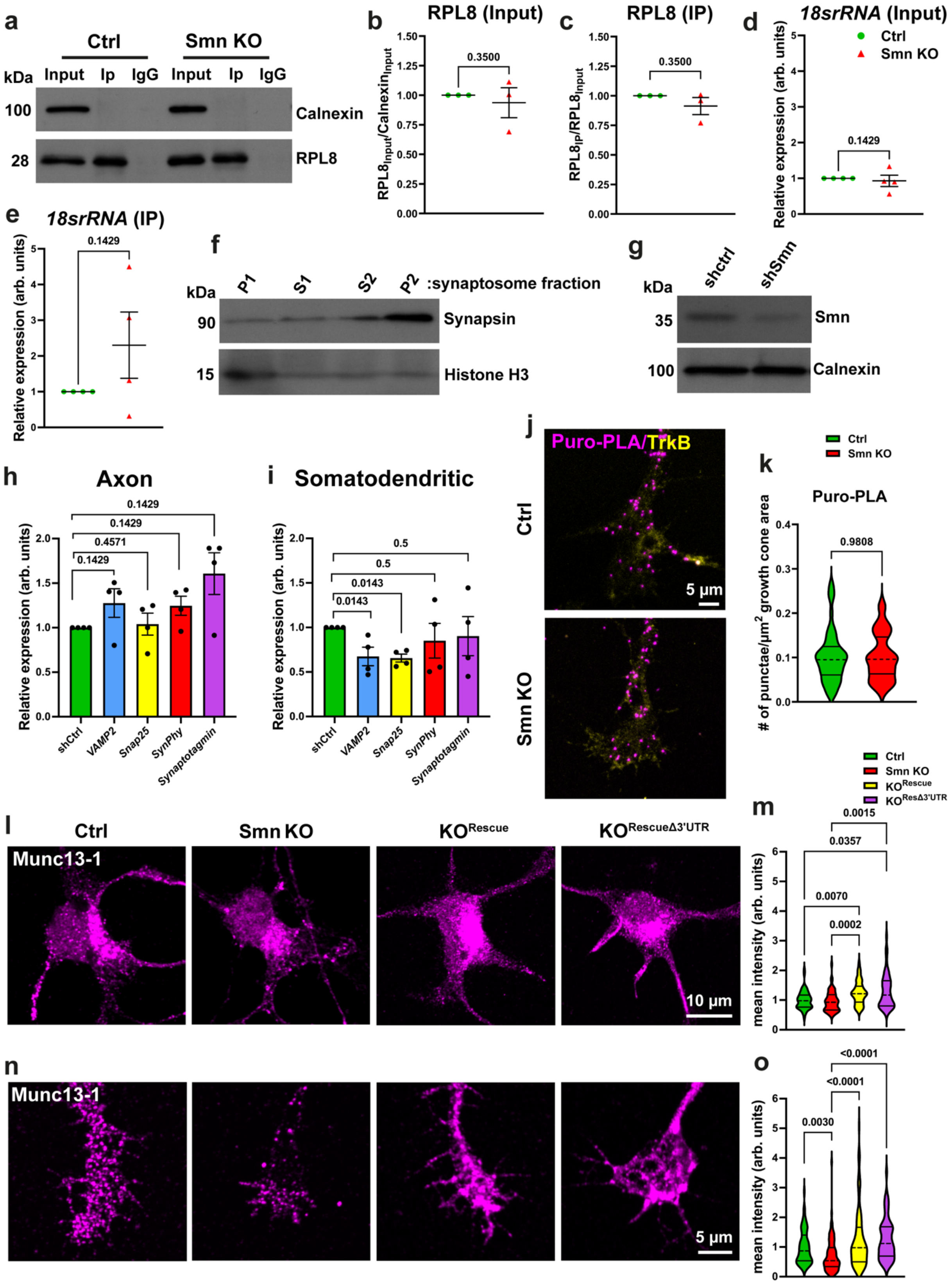

Supplementary Fig. 2 Validation of Munc13-1 Rescue constructs in cultured motoneurons. (**a**) Immunoblot of cortical synaptosomes after RNA immunoprecipitation shows similar ribosome levels in input and immunoprecipitation (IP) fractions of control (Ctrl) and Smn KO

littermates (representative of n = 3 biological replicates). (**b** and **c**) RPL8 levels in input (**b**) and IP (**c**) fractions of cortical synaptosomes are similar between Smn KO and control (n = 3 biological replicates). (**d** and **e**) *18srRNA* levels in input (**d**) and IP (**e**) fractions of cortical synaptosomes are similar between Smn KO and control (n = 4 biological replicates). (**f**) Representative immunoblot of cortical synaptosome fractions showing Synapsin enrichment in synaptosome fractions (P2) compared to Histone H3. (**g**) Immunoblot shows reduced Smn levels in motoneurons after shRNA-mediated knockdown. (**h** and **i**) qRT-PCR analysis reveals unaltered mRNA levels of VAMP2, Snap25, Synaptophysin (SynPhy), and Synaptotagmin in the axonal compartment of Smn knockdown motoneurons. mRNA levels of VAMP2 and Snap25 are slightly reduced in somatodendritic compartments of Smn knockdown motoneurons (*P = 0.0143 from n = 4 biological replicates). (**j**) Representative images of cultured motoneurons showing VAMP2-puromycin-Proximity Ligation Assay (VAMP2-Puro-PLA) signal at axonal growth cones. (**k**) VAMP2-Puro-PLA signal in axonal growth cones is similar between Smn KO and control (n = 52-67 cells from n = 3 biological replicates). (**l**) Representative images of somata of cultured motoneurons stained against Munc13-1. (**m**) Munc13-1 protein levels are increased in somata of Smn KO motoneurons following transduction with Rescue and RescueΔ3'UTR viruses (*P = 0.0357, **P = 0.0015, **P = 0.007, ***P = 0.0002; n = 52-63 cells from n = 3 biological replicates). (**n**) Representative images of axonal growth cones of cultured motoneurons stained against Munc13-1. (**o**) Munc13-1 levels are increased in axonal growth cones of Smn KO motoneurons transduced with Rescue and RescueΔ3'UTR viruses (**P = 0.003, ****P < 0.0001; n = 62-99 cells from n = 3 biological replicates). One-tailed Mann-Whitney U test in **b-i**, Two-tailed Mann-Whitney U test in **k**, and One-way ANOVA with Dunn's post-test in **m** and **o**. Bars represent mean ± SEM. Source data are provided as a Source Data file.

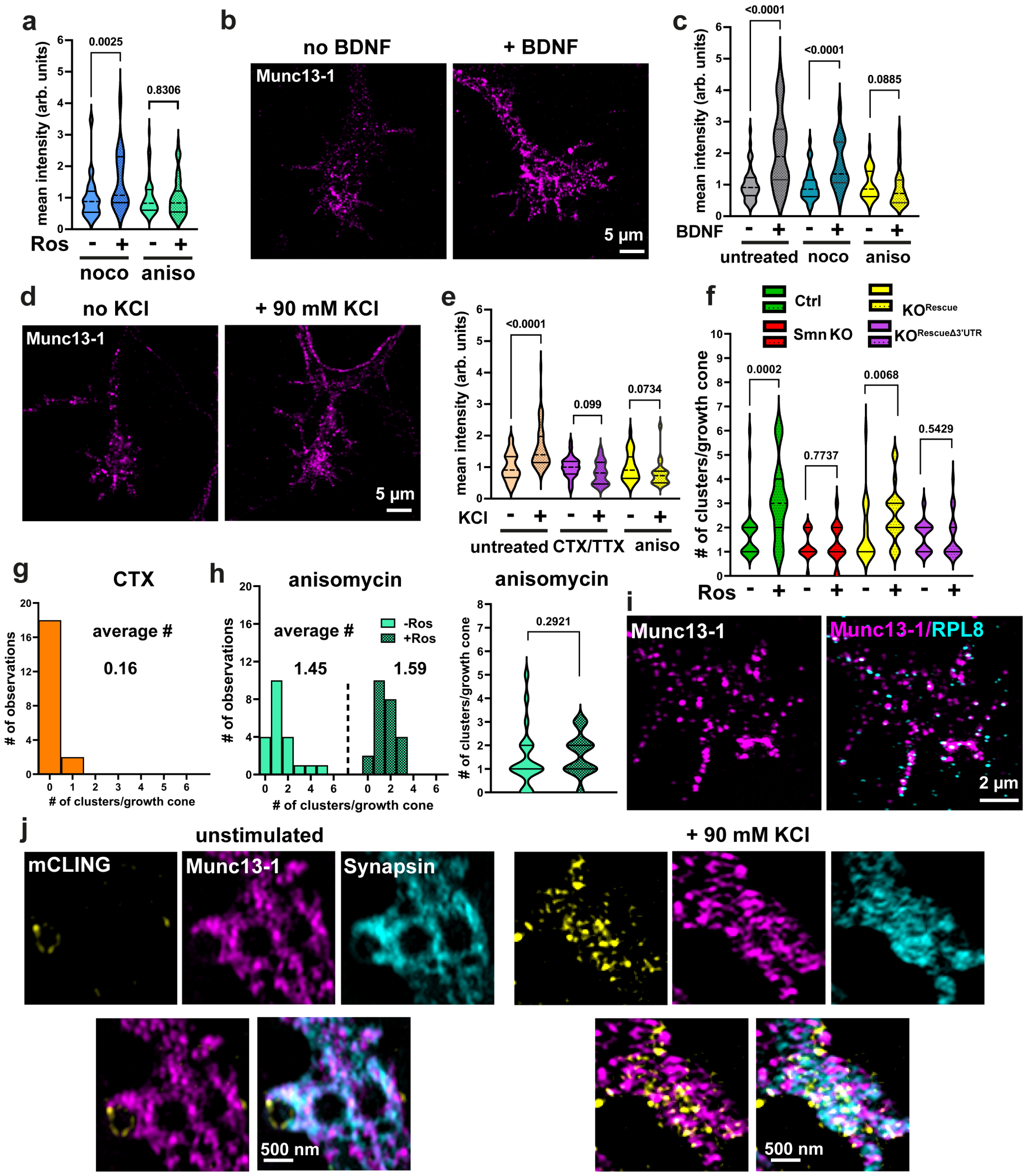

Supplementary Fig. 3 Munc13-1 local translation is triggered by stimulation with BDNF or KCl. (**a**) Nocodazole (noco) treatment does not block Roscovitine (Ros)-induced Munc13-1 local translation (**P = 0.0025; n = 38-42 cells from n = 2 biological replicates), but anisomycin (aniso) does (n = 63 cells from n = 3 biological replicates). (**b**) Representative images of axonal growth cones of cultured motoneurons stained for Munc13-1 following BDNF

stimulation. (**c**) Graph indicates increased Munc13-1 immunoreactivity in axonal growth cones after BDNF stimulation (****P < 0.0001; n = 49-50 cells from n = 2 biological replicates). This increase is also seen after nocodazole (****P < 0.0001; n = 39-40 cells from n = 3 biological replicates), but not after anisomycin treatment (n = 39-41 cells from n = 2 biological replicates). (**d**) Representative images of axonal growth cones of cultured motoneurons stained for Munc13-1 following KCl stimulation. (**e**) Graph reveals increased Munc13-1 immunoreactivity in axonal growth cones after KCl stimulation (****P < 0.0001; n = 63-67 cells from n = 3 biological replicates). This increase is not observed after CTX/TTX (n = 21-29 cells) or anisomycin treatment (n = 20-33 cells) (from n = 1 biological replicate). (**f**) The number of Munc13-1 supramolecular clusters increases significantly in axonal growth cones of Roscovitine-stimulated control and Smn KO^{Rescue} (**P = 0.0068, ***P = 0.0002; n = 21-31 cells from n = 3 biological replicates), but not in stimulated Smn KO and Smn KO$^{Rescue\Delta 3'UTR}$ motoneurons. (**g** and **h)** Treatment with ω-Conotoxin (CTX) (n = 20 cells from n = 1 biological replicate) and anisomycin (n = 21-24 cells from n = 2 biological replicates) inhibits the dynamic formation of Munc13-1 clusters in cultured wt motoneurons. (**i**) Wt motoneurons grown on laminin111 do not exhibit Munc13-1 supramolecular clusters (n = 6 cells from n = 1 biological replicate). (**j**) Representative lattice-SIM maximum projection images of mCLING uptake in growth cones of cultured wt motoneurons (n = 8 cells from n = 1 biological replicate). Upon membrane depolarization, mCLING uptake occurs in close vicinity to Munc13-1 clusters. Two-tailed Mann-Whitney U test in **a-f**. Source data are provided as a Source Data file.

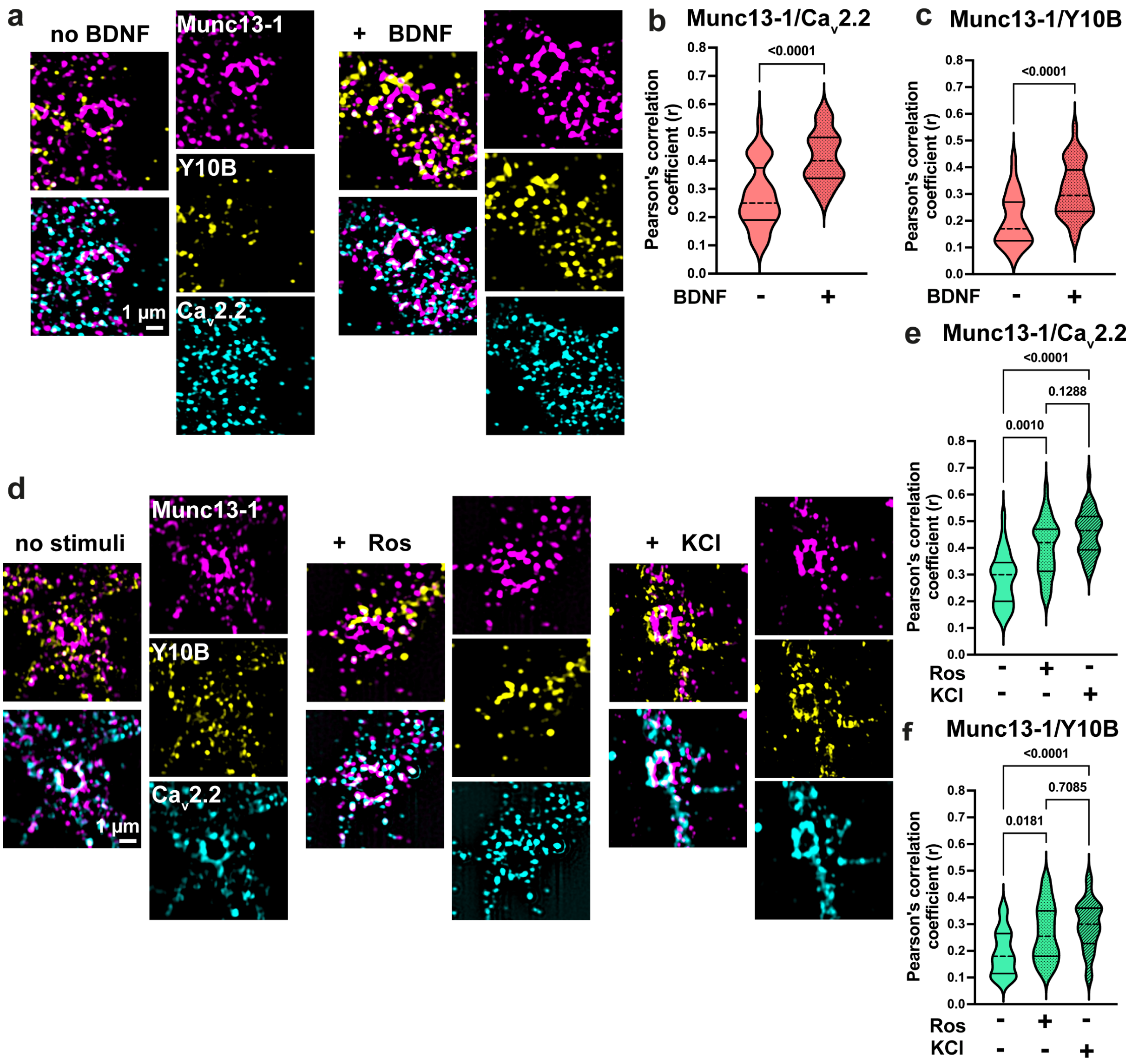

Supplementary Fig. 4 Munc13-1 clusters overlap with Y10B and $Ca_v2.2$ following BDNF or KCl stimulation. (**a**) Representative images of Munc13-1 clusters within axonal growth cones of wt motoneurons following 1-min BDNF pulse stimulation. (**b** and **c**) The colocalization between Munc13-1 clusters and the active zone marker $Ca_v2.2$, as well as the ribosomal marker Y10B, increases significantly upon BDNF stimulation (****P < 0.0001; n = 33-34 cells from n = 2 biological replicates). (**d**) Representative images of Munc13-1 clusters within axonal growth cones of wt motoneurons following 5-min Roscovitine (Ros) or 5-min KCl stimulation. (**e**) The colocalization between Munc13-1 clusters and $Ca_v2.2$ increases significantly upon Roscovitine (***P = 0.001; n = 28-29 cells) or KCl (****P < 0.0001; n = 28-40 cells) stimulation (from n = 2 biological replicates). (**f**) The colocalization between

Munc13-1 and Y10B increases significantly upon Roscovitine (*P = 0.0181; n = 28-29 cells) or KCl (****P < 0.0001; n = 28-40 cells) stimulation (from n = 2 biological replicates). Two-tailed Mann-Whitney U test in **b** and **c**, and One-way ANOVA with Dunn's post-test in **e** and **f**. Source data are provided as a Source Data file.

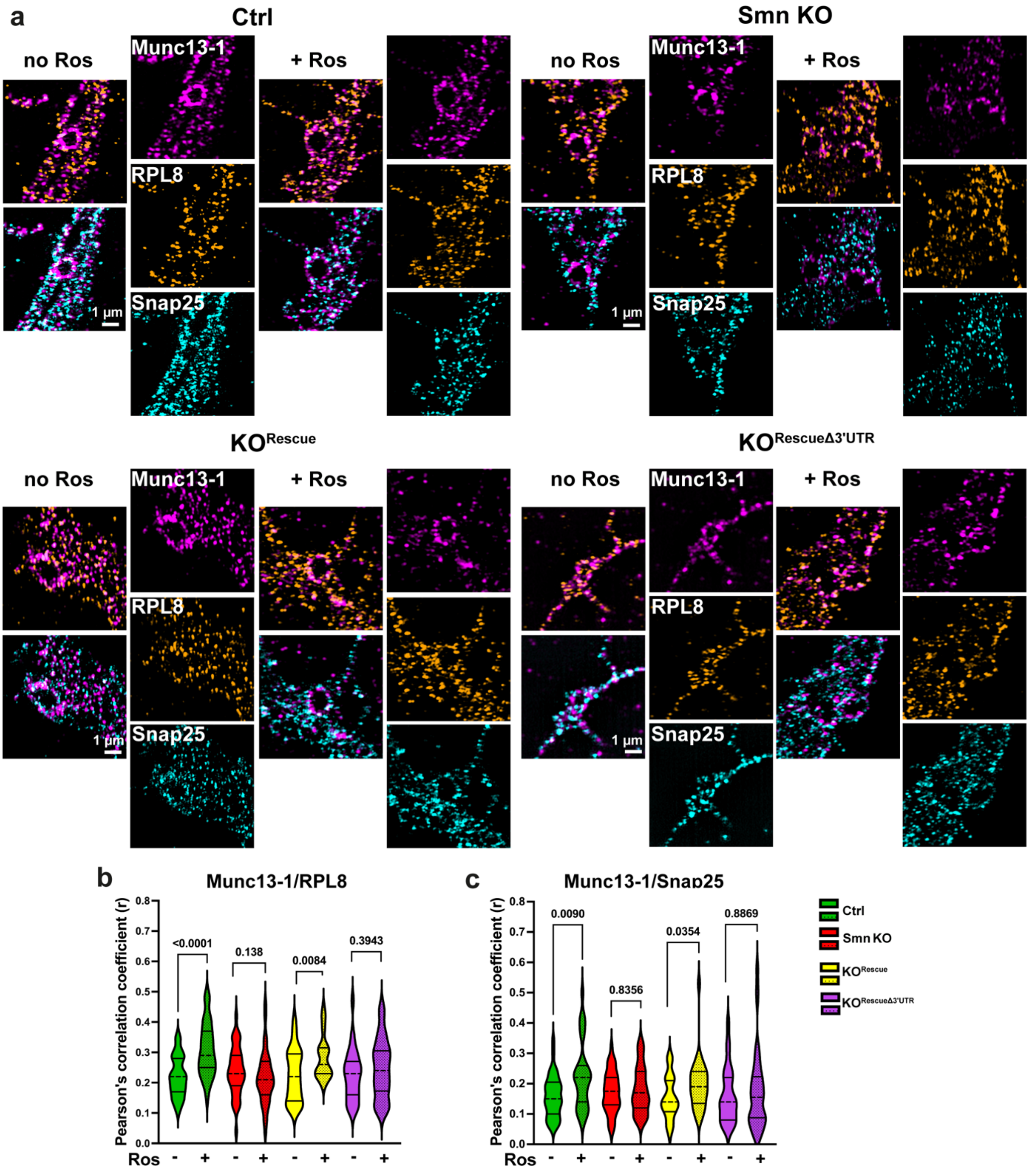

Supplementary Fig. 5 Munc13-1 clusters colocalize with AZ markers in stimulated motoneurons. (**a**) Representative maximum projections of lattice-SIM images indicate Munc13-1 clusters that overlap with RPL8 and Snap25 within axonal growth cones of cultured motoneurons. (**b**) The colocalization between Munc13-1 clusters and RPL8 increases upon Roscovitine (Ros) stimulation in control and Smn KO^{Rescue} motoneurons, but not in Smn KO and Smn KO$^{Rescue\Delta 3'UTR}$ motoneurons (**P = 0.0084, ****P < 0.0001; n = 27-43 cells from n = 3 biological replicates). (**c**) Following Roscovitine stimulation, the colocalization between Munc13-1 clusters and Snap25 increases in control (**P = 0.009; n = 31-36 cells) and Smn KO^{Rescue} motoneurons (*P = 0.0354; n = 29-34 cells), but not in Smn KO (n = 39-40 cells) and KO$^{Rescue\Delta 3'UTR}$ motoneurons (n = 27-38 cells) (from n = 3 biological replicates). Two-tailed Mann-Whitney U test in **b** and **c**. Source data are provided as a Source Data file.

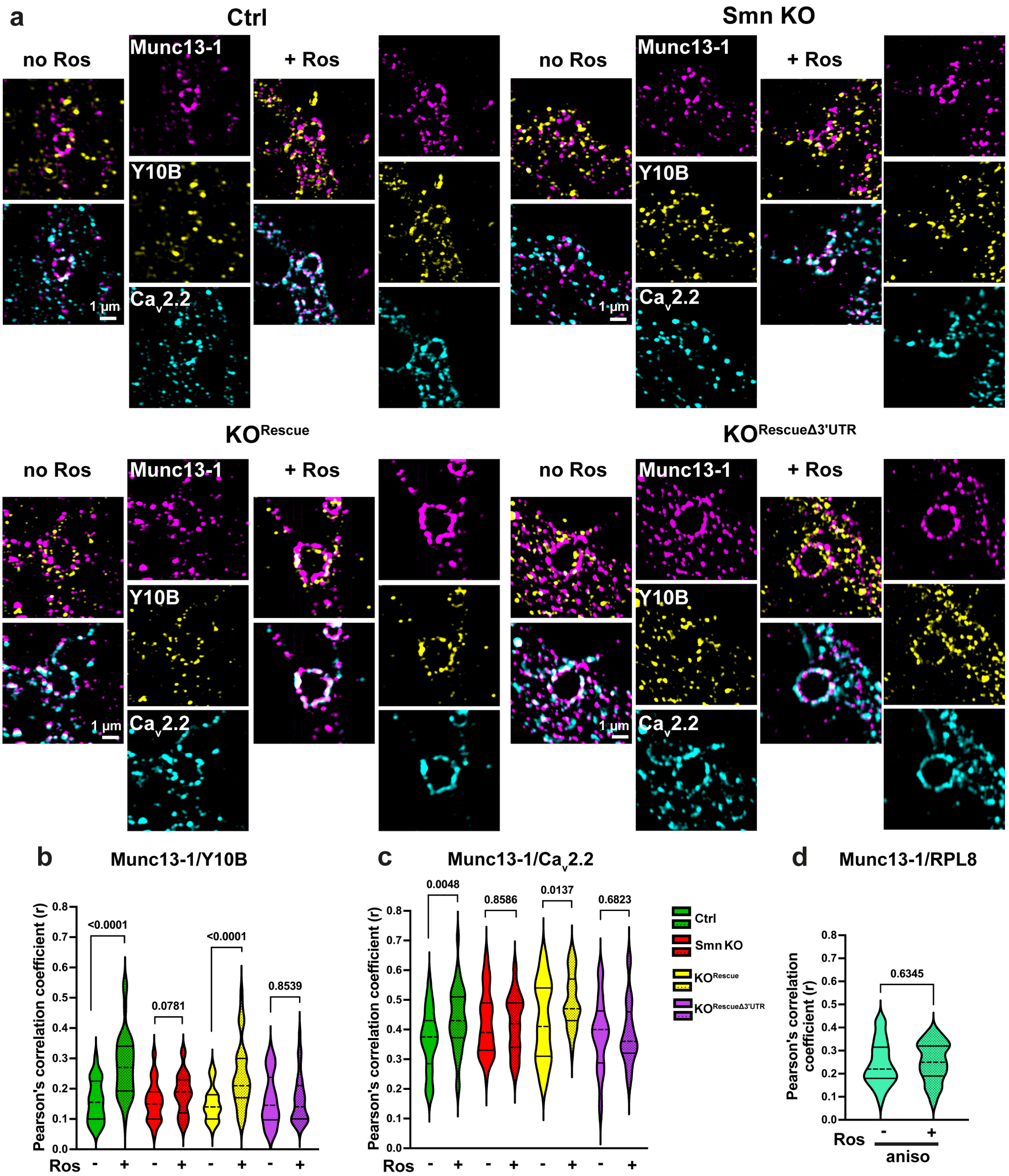

Supplementary Fig. 6 Munc13-1 clusters colocalize with the ribosomal marker, Y10B, in response to stimulation. (**a**) Representative maximum projections of lattice-SIM images indicate Munc13-1 clusters that overlap with Y10B and Ca$_v$2.2 within axonal growth cones of cultured motoneurons. (**b**) Following Roscovitine (Ros) stimulation, the colocalization between Munc13-1 clusters and Y10B increases in control (Ctrl) (****P < 0.0001; n = 36-44 cells) and Smn KO^Rescue motoneurons (****P < 0.0001; n = 41-43 cells), but not in Smn KO (n = 39-40

cells) and KO$^{Rescue\Delta 3'UTR}$ motoneurons (n = 35-38 cells) (from n = 2 biological replicates). (**c**) Following Roscovitine stimulation, the colocalization between Munc13-1 clusters and $Ca_v2.2$ increases in control (**P = 0.0048; n = 36-44 cells) and in Smn KO^{Rescue} motoneurons (*P = 0.0137; n = 41-43 cells), but not in Smn KO (n = 39-41 cells) and KO$^{Rescue\Delta 3'UTR}$ motoneurons (n = 35-38 cells) (from n = 2 biological replicates). (**d**) Anisomycin-treated (aniso) cultured wt motoneurons do not exhibit stimuli-dependent increment in Munc13-1 and RPL8 colocalization in axonal growth cones (n = 23-33 cells from n = 2 biological replicates). Two-tailed Mann-Whitney U test in **b-d**. Source data are provided as a Source Data file.

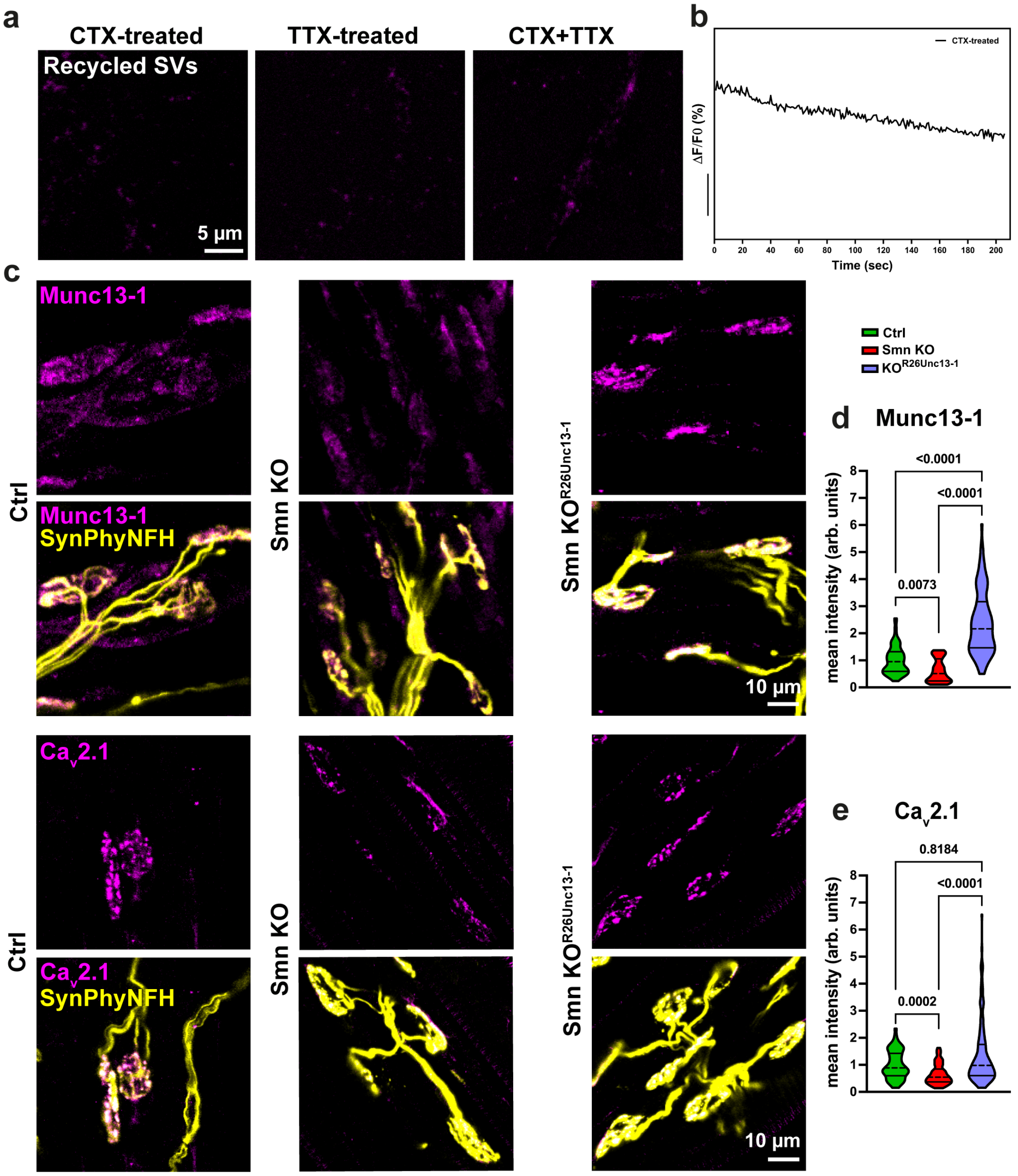

Supplementary Fig. 7 Munc13-1 and Ca$_v$2.1 are upregulated in NMJs of SMA mice expressing the Munc13-1 rescue allele. (**a**) Representative images of SV recycling assay in cultured wt motoneurons treated with ω-Conotoxin (CTX)/Tetrodotoxin (TTX) indicate that the uptake of Synaptotagmin1 antibody depends on neuronal activity (n = 5 cells from n = 1 biological replicate). (**b**) Treatment of motoneurons with CTX prior to Ca^{2+} imaging abolishes the spontaneous Ca^{2+} transients (n = 7 cells from n = 1 biological replicate). (**c**) Representative images of NMJs from P10 animals stained against Munc13-1, Ca$_v$2.1, NFH, and Synaptophysin

(SynPhy). (**d** and **e**) Munc13-1 (**P = 0.0073, ****P < 0.0001; n = 55-112 NMJs) and $Ca_v2.1$ (***P = 0.0002, ****P < 0.0001; n = 59-159 NMJs) are upregulated in NMJs of TVA muscles from Smn $KO^{R26Unc13\text{-}1tg/+}$ (n = 3 biological replicates) compared to control (Ctrl) and Smn KO littermates (n = 2 biological replicates). One-way ANOVA with Dunn's post-test in **d** and **e**. Source data are provided as a Source Data file.

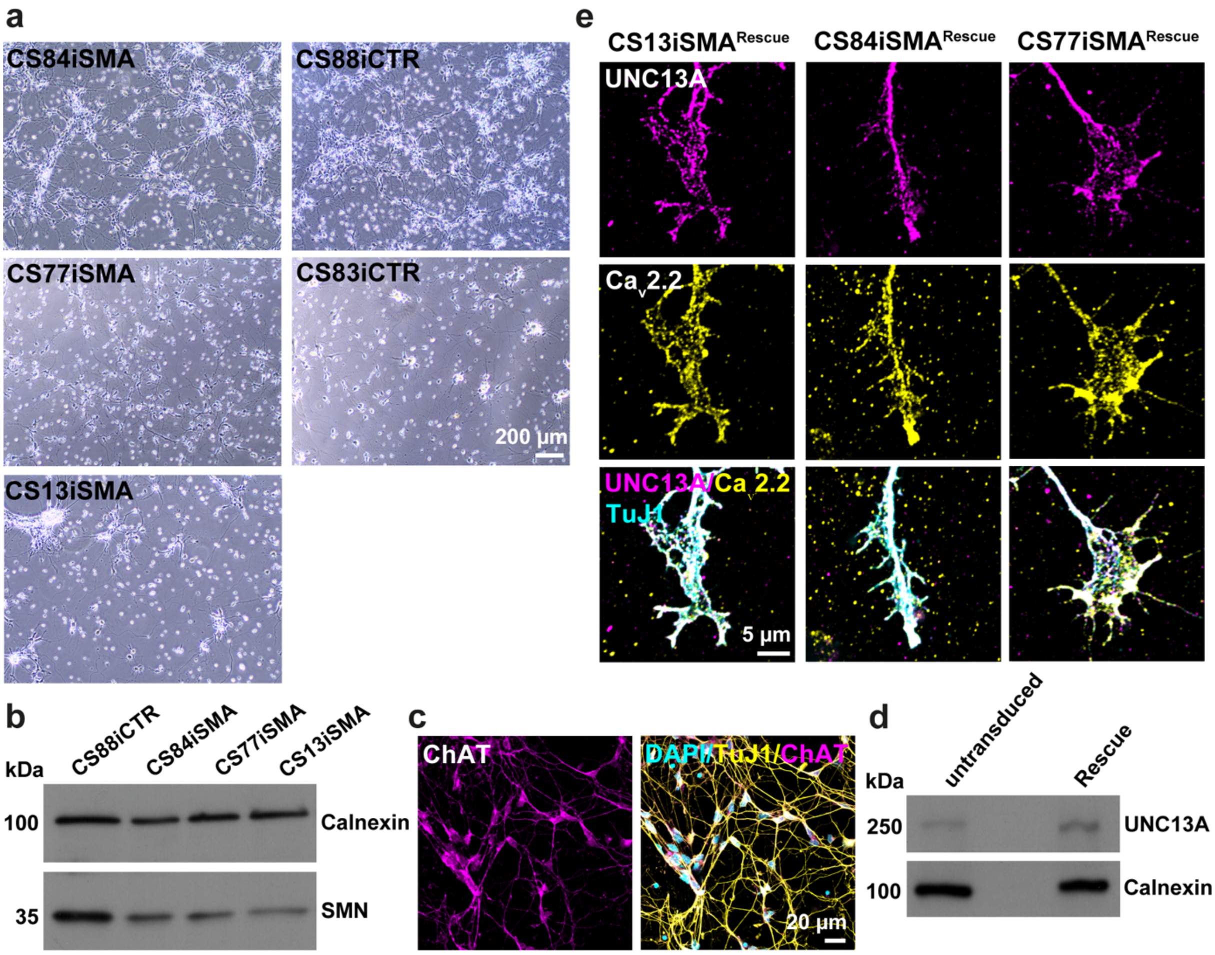

Supplementary Fig. 8 Characterization of human iPSC-derived motoneurons from SMA patients and control individuals. (a) Representative images of cultured DIV25 hiPSC-derived motoneurons from three SMA patients and two control individuals. (**b**) Immunoblot of total lysates obtained from hiPSCs reveals reduced SMN levels in SMA-hiPSCs compared to control. (**c**) Representative images of cultured DIV25 hiPSC-derived motoneurons expressing ChAT and TuJ1. Nuclei are stained with DAPI. (**d**) Immunoblot shows increased UNC13A protein levels in total lysates from human control motoneurons transduced with Rescue

lentivirus expressing UNC13A+SYP3'UTR (representative of n = 3 biological replicates). (**e**) Representative images of axonal growth cones of cultured DIV25 hiPSC-derived motoneurons from three SMA patients transduced with UNC13A+SYP3'UTR Rescue virus stained against TuJ1, UNC13A, and $Ca_v2.2$ (related to Fig. 9, c and d). Source data are provided as a Source Data file.

Supplementary Table 1

Gene	In somatodendritic compartment: # of reads				In axonal compartment: # of reads			
	Control	shSmn	log2 (fold change)	p-value	Control	shSmn	log2 (fold change)	p-value
Vamp2	148.774	374.628	1.3323	0.00005	40.8212	151.603	1.8929	0.00005
Scamp5	46.7639	99.3003	1.0864	0.00005	15.584	49.6606	1.6720	0.00005
Stx1	20.9149	43.4892	1.0561	0.00045	35.851	55.0863	0.6196	0.05545
Syp	136.265	231.251	0.7630	0.0024	37.9882	143.575	1.9181	0.0001
Syt1	35.7827	56.5509	0.6602	0.0007	14.6472	32.0191	1.1283	0.0107
Sv2a	41.5968	64.9948	0.6438	0.0004	13.5468	28.5401	1.0750	0.00605
Vat1	44.2847	64.8327	0.5499	0.00415	11.8737	38.1125	1.6824	0.00165
Snap25	190.679	275.378	0.5302	0.0026	58.4401	189.062	1.6938	0.00005
Syt5	23.7289	28.7549	0.2771	0.2948	10.7778	31.8084	1.5613	0.00215
Snap91	62.1812	65.5011	0.0750	0.6654	13.3078	31.5119	1.2436	0.001
Stx7	32.616	40.0225	0.29523	0.1438	11.7152	27.3224	1.2217	0.003

Supplementary Table 1. List of transcripts of synaptic proteins that are upregulated in axons of Smn-knockdown motoneurons. Transcripts for some synaptic proteins are upregulated in the somata and axons of Smn-knockdown motoneurons compared to control as determined by RNAseq. Transcripts for Snap91, Syt5, and Stx7 are upregulated only in the axonal compartment (table is prepared from previously published RNAseq data [1]).

Supplementary References

1 Hennlein, L. *et al.* Plastin 3 rescues cell surface translocation and activation of TrkB in spinal muscular atrophy. *The Journal of cell biology* **222** (2023). https://doi.org/10.1083/jcb.202204113
