# Supplementary material for: Munc13-1 restoration mitigates presynaptic pathology in spinal muscular atrophy
Source: Nat Commun. 2025 Sep 30;16:8724. doi: 10.1038/s41467-025-64164-w (PMC12485113; doi:10.1038/s41467-025-64164-w)
Supplement: Supplementary file 2 — Description of Additional Supplementary Files [file 41467_2025_64164_MOESM2_ESM.pdf]

Description of Additional Supplementary Files

Supplementary Movie 1: Munc13-1 nanoassemblies in presynaptic membranes of unstimulated motoneurons. Videos of z-stacks from expanded axonal growth cones of unstimulated cultured wt motoneurons from Fig. 4a, showing Munc13-1 nanoassemblies (magenta). Images were acquired by lattice-SIM. Scale bar: 1 µm.

Supplementary Movie 2: Munc13-1 nanoassemblies in presynaptic membranes of stimulated motoneurons. Videos of z-stacks from expanded axonal growth cones in Roscovitine-stimulated cultured wt motoneurons from Fig. 4a, showing Munc13-1 nanoassemblies (magenta). Images were acquired by lattice-SIM. Scale bar: 1 µm.
