# Supplementary material for: Munc13-1 restoration mitigates presynaptic pathology in spinal muscular atrophy
Source: Nat Commun. 2025 Sep 30;16:8724. doi: 10.1038/s41467-025-64164-w (PMC12485113; doi:10.1038/s41467-025-64164-w)
Supplement: Supplementary file 5 — Reporting Summary [file 41467_2025_64164_MOESM5_ESM.pdf]

nature portfolio

Corresponding author(s): Dr. Mehri Moradi and Dr. Michael Sendtner

Last updated by author(s): Sep 2, 2025

Reporting Summary

Nature Portfolio wishes to improve the reproducibility of the work that we publish. This form provides structure for consistency and transparency in reporting. For further information on Nature Portfolio policies, see our Editorial Policies and the Editorial Policy Checklist.

Statistics

For all statistical analyses, confirm that the following items are present in the figure legend, table legend, main text, or Methods section.

n/a	Confirmed	
☐	☒	The exact sample size (*n*) for each experimental group/condition, given as a discrete number and unit of measurement
☐	☒	A statement on whether measurements were taken from distinct samples or whether the same sample was measured repeatedly
☐	☒	The statistical test(s) used AND whether they are one- or two-sided<br>*Only common tests should be described solely by name; describe more complex techniques in the Methods section.*
☐	☒	A description of all covariates tested
☐	☒	A description of any assumptions or corrections, such as tests of normality and adjustment for multiple comparisons
☐	☒	A full description of the statistical parameters including central tendency (e.g. means) or other basic estimates (e.g. regression coefficient) AND variation (e.g. standard deviation) or associated estimates of uncertainty (e.g. confidence intervals)
☐	☒	For null hypothesis testing, the test statistic (e.g. *F*, *t*, *r*) with confidence intervals, effect sizes, degrees of freedom and *P* value noted<br>*Give P values as exact values whenever suitable.*
☒	☐	For Bayesian analysis, information on the choice of priors and Markov chain Monte Carlo settings
☒	☐	For hierarchical and complex designs, identification of the appropriate level for tests and full reporting of outcomes
☐	☒	Estimates of effect sizes (e.g. Cohen's *d*, Pearson's *r*), indicating how they were calculated

Our web collection on statistics for biologists contains articles on many of the points above.

Software and code

Policy information about availability of computer code

Data collection	No software was used for Data collection
Data analysis	GraphPad Prism (version 10), ImageJ (version 1.54f), Nikon Element image software, commercial software package from Zeiss (ZEN 3.0 SR FP2 black), ZEN 2 blue software (Zeiss, version 3.5).

For manuscripts utilizing custom algorithms or software that are central to the research but not yet described in published literature, software must be made available to editors and reviewers. We strongly encourage code deposition in a community repository (e.g. GitHub). See the Nature Portfolio guidelines for submitting code & software for further information.

Data

Policy information about availability of data

All manuscripts must include a data availability statement. This statement should provide the following information, where applicable:

- Accession codes, unique identifiers, or web links for publicly available datasets
- A description of any restrictions on data availability
- For clinical datasets or third party data, please ensure that the statement adheres to our policy

All datasets are available in the publicly accessible repository "Figshare" under the following accession code: https://doi.org/10.6084/m9.figshare.29971474. Source Data are provided with this paper.

Research involving human participants, their data, or biological material

Policy information about studies with human participants or human data. See also policy information about sex, gender (identity/presentation), and sexual orientation and race, ethnicity and racism.

Reporting on sex and gender	N/A
Reporting on race, ethnicity, or other socially relevant groupings	N/A
Population characteristics	N/A
Recruitment	N/A
Ethics oversight	N/A

Note that full information on the approval of the study protocol must also be provided in the manuscript.

Field-specific reporting

Please select the one below that is the best fit for your research. If you are not sure, read the appropriate sections before making your selection.

[x] Life sciences [ ] Behavioural & social sciences [ ] Ecological, evolutionary & environmental sciences

For a reference copy of the document with all sections, see nature.com/documents/nr-reporting-summary-flat.pdf

Life sciences study design

All studies must disclose on these points even when the disclosure is negative.

Sample size	Most experiments were carried out independently at least in triplicate. No calculations were done to predetermine sample size. The sample size was chosen based on the author's experience and established standards for mouse tissues, cell lines, primary motoneuron cultures, and human induced pluripotent stem cells (iPSCs). All attempts at replication were successful. For exploratory experiments, sample sizes were chosen to ensure reproducibility across independent replicates and to capture variability within the system. For confirmatory experiments, the number of biological replicates was determined based on prior experience with similar assays, where these sample sizes have reliably provided robust and interpretable results. We selected group sizes that are consistent with established standards in the field and sufficient to detect biologically meaningful differences while minimizing unnecessary use of resources.
Data exclusions	No data were excluded from experiments with cultured motoneurons, hiPSC cells or mouse tissues. No animals were excluded from motor assessments. Two litters, out of 29, were excluded from the survival assessment, wherein the female stopped to suckle the litter leading to the death of all pubs. In addition, pubs, which died at P0, were not considered for the survival and motor assessment.
Replication	To ensure reproducibility, most experiments were carried out at least in triplicate.
Randomization	All experiments were conducted using primary motoneurons, tissues from genetically defined mice, the NSC34 cell line, and human iPSCs. Treatment conditions and controls were selected from the same batch to ensure consistency. Additionally, blinding was applied during data collection and analysis to further reduce potential bias in outcome interpretation.
Blinding	All immunostaining experiments, including immunocytochemistry (ICC), immunohistochemistry (IHC), single-molecule fluorescence in situ hybridization (smFISH), and proximity ligation assays (PLA), were carried out and analyzed in a blinded manner, except for ICC in super-resolution microscopy. Similarly, all animal behaviour experiments, including motor assessments and survival studies, were performed and analyzed with blinding to minimize observer bias. Experimenters were blinded to group assignments during data collection and analysis to ensure objective and unbiased interpretation of results.

Reporting for specific materials, systems and methods

We require information from authors about some types of materials, experimental systems and methods used in many studies. Here, indicate whether each material, system or method listed is relevant to your study. If you are not sure if a list item applies to your research, read the appropriate section before selecting a response.

Materials & experimental systems

n/a	Involved in the study
☐	☒ Antibodies
☐	☒ Eukaryotic cell lines
☒	☐ Palaeontology and archaeology
☐	☒ Animals and other organisms
☒	☐ Clinical data
☒	☐ Dual use research of concern
☒	☐ Plants

Methods

n/a	Involved in the study
☒	☐ ChIP-seq
☒	☐ Flow cytometry
☒	☐ MRI-based neuroimaging

Antibodies

Antibodies used

Used dyes:
1) mCLING-ATTO 647N (Synaptic Systems, 710006AT1)
2) alpha-Bungarotoxin (ThermoFisher Scientific, B13422, 1:1000)
3) calcium indicator Oregon GreenTM 488 BAPTA-1, AM, cell-permeant (ThermoFisher Scientific, O6807)
4) Alexa Fluor 488 NHS diluted (ThermoFisher, A20000, 20 µg/ml)

Primary antibodies:
1) rabbit polyclonal anti-Tau (Sigma-Aldrich, T6402, 1:1000)
2) mouse monoclonal anti-α-Tubulin, clone AA13 (Sigma-Aldrich, T5168, 1:1000)
3) mouse monoclonal purified IgG anti-Basoon (Synaptic Systems, 141011, 1:500)
4) guinea pig polyclonal antiserum anti-Piccolo (Synaptic systems, 142104, 1:500)
5) rabbit polyclonal purified anti-RIM1/2 (Synaptic Systems, 140213, 1:500)
6) rabbit polyclonal anti-Munc13-1 (Synaptic System, 126103, 1:500)
7) guinea pig polyclonal purified anti-Ca2+ channel N-type alpha-1B (Cav2.2) (Synaptic System, 152305, 1:250)
8) goat polyclonal anti-ribosomal protein L8 (RPL8) (Sigma-Aldrich, SAB2500882, 1:500)
9) guinea pig monoclonal recombinant IgG anti-Snap25 (Synaptic systems, 111308, 1:250)
10) guinea pig polyclonal antiserum anti-Synapsin1/2 (Synaptic systems, 111308, 1:500)
11) goat anti-Choline Acetyltransferase (Millipore, AB144P, 1:250)
12) anti-TuJ1 (Neuromics, MO15013, 1:1000)
13) polyclonal goat anti-TrkB (Bio-Techne Sales Corp, AF1494, 1:500)
14) guinea pig polyclonal anti-Synaptophysin1 (Synaptic Systems, 101004, 1:1000)
15) rabbit polyclonal anti-Ca2+ channel P/Q-type specific against the alpha-1A subunit (Cav2.1) (Synaptic Systems, 152203, 1:500)
16) guinea pig polyclonal antiserum anti-Munc13-1 (Synaptic Systems, 126104, 1:500)
17) chicken polyclonal anti-Neurofilament H (Merck, AB5539, 1:1000)
18) Synaptotagmin1 antibody luminal domain (Synaptic Systems, 105103C3)
19) rabbit polyclonal anti-Calnexin (Enzo Life Sciences, ADI-SPA-860-F, 1:6000)
20) mouse monoclonal anti-SMN (BD Biosciences, 610646, 1:5000)
21) rabbit polyclonal anti-Cre (Merck, 69050, 1:5000)
22) mouse monoclonal anti-β-actin (GeneTex, GTX26276, 1:5000)
23) rabbit polyclonal anti-Synaptobrevin2 (VAMP2) (Synaptic System, 104008, 1:500)
24) rabbit polyclonal anti-Histone H3 (Abcam, ab1791, 1:10000)

Secondary antibodies:
1) donkey anti-mouse IgG (H+L) (Alexa Fluor 488, Jackson ImmunoResearch, 715-545-150)
2) donkey anti-rabbit IgG (H+L) AffiniPure (Alexa Fluor 488, Jackson ImmunoResearch, 711-545-152)
3) donkey anti-rabbit IgG (H+L) AffiniPure (Cy3, Jackson ImmunoResearch, 711-165-152)
4) donkey F(ab')2 anti-rabbit IgG (H+L) (Alexa Fluor 647, Abcam, ab181347, 1:300)
5) donkey anti guinea pig (Alexa Fluor 488, Dianova, 140967, 1:300)
6) donkey anti-guinea pig IgG (H+L) AffiniPure (Cy5, Jackson ImmunoResearch, 706-175-148)
7) donkey anti-goat IgG (H+L) AffiniPure (Cy3, Jackson ImmunoResearch, 705-165-147, 1:500)
8) donkey anti-goat IgG (H+L) (CF568, Biotium, 20106, 1:500)
9) donkey anti-goat IgG (H+L) AffiniPure (Alexa Fluor 647, Jackson ImmunoResearch, 705-605-003)
10) donkey anti-chicken IgY (H+L) AffiniPure (Cy5, Jackson ImmunoResearch, 703-175-155, 1:500)
11) peroxidase AffiniPure donkey anti-mouse IgG (H +L) (Biozol, 715-035-151, 1:10000)
12) peroxidase AffiniPure goat anti-rabbit IgG (H +L) (Biozol, 111-035-144, 1:10000)
13) peroxidase AffiniPure donkey anti-goat IgG (H +L) (Biozol, 705-035-003, 1:10000)

Validation

Primary antibodies:
1) rabbit polyclonal anti-Tau (Sigma-Aldrich, T6402, 1:1000). This antibody is reported to react with mouse and rat (https://www.sigmaaldrich.com/DE/de/product/sigma/t6402?srsltid=AfmBOoocovZE4QZHdmjA85fGdyFxRvULALC1hszWpEGrOn0p_Nsv2Aw-#product-documentation).
2) mouse monoclonal anti-α-Tubulin, clone AA13 (Sigma-Aldrich, T5168, 1:1000). This antibody is reported to react with

mouse,chicken,Chlamydomonas,Africangreenmonkey,human,rat,bovine,seaurchin,kangaroorat(https://www.sigmaaldrich.com/DE/de/product/sigma/t5168)
3) mousemonoclonalpurifiedIgGanti-Basoon(SynapticSystems,141011,1:500).Thisantibodyisreportedtoreactwithmouseandrat (https://www.sysy.com/product/141003).
4) guineapigpolyclonalantiserumanti-Piccolo(Synapticsystems,142104,1:500).ValidatedbythesupplierusingKOrats(https://www.sysy.com/product/142104).
5) rabbitpolyclonalpurifiedanti-RIM1/2(SynapticSystems,140213,1:500). This antibody is reported to react with mouse and rat (https://www.sysy.com/product/140213).
6) rabbit polyclonal anti-Munc13-1 (Synaptic System, 126103, 1:500). Validated by the supplier using KO mice (https://www.sysy.com/product/126103).
7) guinea pig polyclonal purified anti-Ca2+ channel N-type alpha-1B (Cav2.2) (Synaptic System, 152305, 1:250). This antibody is reported to react with mouse and rat (https://www.sysy.com/product/152305).
8) goat polyclonal anti-ribosomal protein L8 (RPL8) (Sigma-Aldrich, SAB2500882, 1:500). Validation data provided by the supplier.
9) guinea pig monoclonal recombinant IgG anti-Snap25 (Synaptic systems, 111308, 1:250). Validated by the supplier using KO mice (https://www.sysy.com/product/111308).
10) guinea pig polyclonal antiserum anti-Synapsin1/2 (Synaptic systems, 111308, 1:500). Validated by the supplier using KO mice (https://www.sysy.com/product/111308).
11) goat anti-Choline Acetyltransferase (Millipore, AB144P, 1:250). Validation data provided by the supplier (https://www.merckmillipore.com/DE/de/product/Anti-Choline-Acetyltransferase-Antibody,MM_NF-AB144P).
12) anti-TuJ1 (Neuromics, MO15013, 1:1000). Validation data provided by the supplier (https://www.neuromics.com/ittrium/reference/D8x1b63x8x1).
13) polyclonal goat anti-TrkB (Bio-Techne Sales Corp, AF1494, 1:500). Validated in our lab using KO mice (https://doi.org/10.1186/s40035-022-00304-2).
14) guinea pig polyclonal anti-Synaptophysin1 (Synaptic Systems, 101004, 1:1000). This Product has been discontinued. No validation is available.
15) rabbit polyclonal anti-Ca2+ channel P/Q-type specific against the alpha-1A subunit (Cav2.1) (Synaptic Systems, 152203, 1:500). Validated by the supplier using KO mice (https://www.sysy.com/product/152203).
16) guinea pig polyclonal antiserum anti-Munc13-1 (Synaptic Systems, 126104, 1:500). Validated by the supplier using KO mice (https://www.sysy.com/product/126104).
17) chicken polyclonal anti-Neurofilament H (Merck, AB5539, 1:1000). Validation data provided by the supplier (https://www.merckmillipore.com/DE/de/product/Anti-Neurofilament-H-Antibody,MM_NF-AB5539#anchor_Product%20Information).
18) Synaptotagmin1 antibody luminal domain (Synaptic Systems, 105103C3). This antibody is reported to react with mouse and rat (https://www.sysy.com/product/105103C3).
19) rabbit polyclonal anti-Calnexin (Enzo Life Sciences, ADI-SPA-860-F, 1:6000). Validated in our lab using Calnexin KO mice (https://doi.org/10.1016/j.devcel.2023.07.004).
20) mouse monoclonal anti-SMN (BD Biosciences, 610646, 1:5000). Validated in our lab using KO mice (https://doi.org/10.1186/s40035-022-00304-2).
21) rabbit polyclonal anti-Cre (Merck, 69050, 1:5000). Validated in our lab using Cre transgenic mice and transfection with Cre-expressing viruses.
22) mouse monoclonal anti-β-actin (GeneTex, GTX26276, 1:5000). Validated in our lab using β-actin Knockdown tools (https://doi.org/10.1083/jcb.201604117).
23) rabbit polyclonal anti-Synaptobrevin2 (VAMP2) (Synaptic System, 104008, 1:500). Validated by the supplier using KO mice. (https://www.sysy.com/product/104008).
24) rabbit polyclonal anti-Histone H3 (Abcam, ab1791, 1:10000), Validated by the supplier (https://www.abcam.com/en-us/products/primary-antibodies/histone-h3-antibody-nuclear-marker-and-chip-grade-ab1791#).

Secondary antibodies:
1) donkey anti-mouse IgG (H+L) (Alexa Fluor 488, Jackson ImmunoResearch, 715-545-150). Validated in our lab by omission of primary antibody, absence of target antigen and used under similar conditions for other projects.
2) donkey anti-rabbit IgG (H+L) AffiniPure (Alexa Fluor 488, Jackson ImmunoResearch, 711-545-152). Validated in our lab by omission of primary antibody, absence of target antigen and used under similar conditions for other projects.
3) donkey anti-rabbit IgG (H+L) AffiniPure (Cy3, Jackson ImmunoResearch, 711-165-152). Validated in our lab by omission of primary antibody, absence of target antigen and used under similar conditions for other projects.
4) donkey F(ab')2 anti-rabbit IgG (H+L) (Alexa Fluor 647, Abcam, ab181347, 1:300). Validated in our lab by omission of primary antibody, absence of target antigen and used under similar conditions for other projects.
5) donkey anti guinea pig (Alexa Fluor 488, Dianova, 140967, 1:300). Validated in our lab by omission of primary antibody, absence of target antigen and used under similar conditions for other projects.
6) donkey anti-guinea pig IgG (H+L) AffiniPure (Cy5, Jackson ImmunoResearch, 706-175-148). Validated in our lab by omission of primary antibody, absence of target antigen and used under similar conditions for other projects.
7) donkey anti-goat IgG (H+L) AffiniPure (Cy3, Jackson ImmunoResearch, 705-165-147, 1:500). Validated in our lab by omission of primary antibody, absence of target antigen and used under similar conditions for other projects.
8) donkey anti-goat IgG (H+L) (CF568, Biotium, 20106, 1:500). Validated in our lab by omission of primary antibody, absence of target antigen and used under similar conditions for other projects.
9) donkey anti-goat IgG (H+L) AffiniPure (Alexa Fluor 647, Jackson ImmunoResearch, 705-605-003). Validated in our lab by omission of primary antibody, absence of target antigen and used under similar conditions for other projects.
10) donkey anti-chicken IgY (H+L) AffiniPure (Cy5, Jackson ImmunoResearch, 703-175-155, 1:500). Validated in our lab by omission of primary antibody, absence of target antigen and used under similar conditions for other projects.
11) peroxidase AffiniPure donkey anti-mouse IgG (H +L) (Biozol, 715-035-151, 1:10000). Validated in our lab by omission of primary antibody, absence of target antigen and used under similar conditions for other projects.
12) peroxidase AffiniPure goat anti-rabbit IgG (H +L) (Biozol, 111-035-144, 1:10000). Validated in our lab by omission of primary antibody, absence of target antigen and used under similar conditions for other projects.
13) peroxidase AffiniPure donkey anti-goat IgG (H +L) (Biozol, 705-035-003, 1:10000). Validated in our lab by omission of primary

antibody, absence of target antigen and used under similar conditions for other projects.

Eukaryotic cell lines

Policy information about cell lines and Sex and Gender in Research

Cell line source(s)	NSC-34 cells (Cedarlane, cat. no. CLU140)
Authentication	NSC-34 cells were obtained commercially and were not authenticated.
Mycoplasma contamination	NSC-34 cells tested negative for mycoplasma contamination.
Commonly misidentified lines (See ICLAC register)	No commonly misidentified cell lines were used in the study.

Animals and other research organisms

Policy information about studies involving animals; ARRIVE guidelines recommended for reporting animal research, and Sex and Gender in Research

Laboratory animals	Laboratory mice were housed in the local animal facility following the regulations on animal protection of the national federal law and of the Association for Assessment and Accreditation of Laboratory Animal care. Mice were housed under controlled conditions on a 12 h light/12 h dark cycle at 20–22 °C and 55–65% relative humidity, with ad libitum access to food and water. SMA litters, Smn-/-,Hungtg/+ and control litters,Smn+/-,Hungtg/+ were offspring of two mouse strains (I) Smn+/-that is hemizygote for the Smntm1Hung targeted mutation, and (II) Smn-/-,Hungtg/tg that is homozygote for the Smntm1Hung targeted mutation as well as for the transgenic Hung allele, Tg(SMN2)2Hung. Both mouse lines were obtained from Jackson Repository and maintained on a C57BL/6J background (C57BL/6J.29P2-Smn1Hung<tm1Msd>/J).C57BL/6J mice (referred to as " wt " in the text) were used for all control experiments as well as for expansion microscopy and were obtained from Charles River Repository. Munc13-1 KO mice (Munc13-1-/-) were originally obtained from Göttingen, Germany, and cross-bred in-house. Nestin-Cre transgenic mice(C57BL/6J.Cg(Nes-cre)1Kln/J) were cross-bred in-house. The R26Unc13-1tg/+ knock-in mouse model was generated at the Czech Centre for Phenogenomics in Prague, Czech Republic (https://www.phenogenomics.cz). Smn+/-,R26Unc13-1tg/+ and Smn-/-,Hungtg/tg,Nestin-Cretg/+ mice were cross-bred from parents and generated in-house. For primary motoneuron cultures, embryos were obtained from pregnant mice at embryonic day 12.5 (E12.5). For immunohistochemistry, mice were used at postnatal day 5 (P5) or 10 (P10). For the RNA immunoprecipitation assay, mice were used at postnatal day 5 (P5). For behavioral experiments, mice were used at postnatal day 10 (P10).
Wild animals	Wild animals were not used for this study.
Reporting on sex	Sex was not considered regarding embryonic mice used for motoneuron cultures. Animals for tissue harvesting and motor behavioural testing were not discriminated between sex and were used equally.
Field-collected samples	This study did not involve samples collected from the field.
Ethics oversight	All animal experiments were performed strictly according to the regulations on animal protection of the German federal law and the Association of Assessment and Accreditation of Laboratory Animal Care, in agreement with and under the control of the local veterinary authority. This study was approved under protocol number 55.2.2-2532.2-924-14.

Note that full information on the approval of the study protocol must also be provided in the manuscript.

Plants

Seed stocks	N/A
Novel plant genotypes	N/A
Authentication	N/A
