# Supplementary figures and images for: Munc13-1 restoration mitigates presynaptic pathology in spinal muscular atrophy
Source: Nat Commun. 2025 Sep 30;16:8724. doi: 10.1038/s41467-025-64164-w (PMC12485113; doi:10.1038/s41467-025-64164-w)

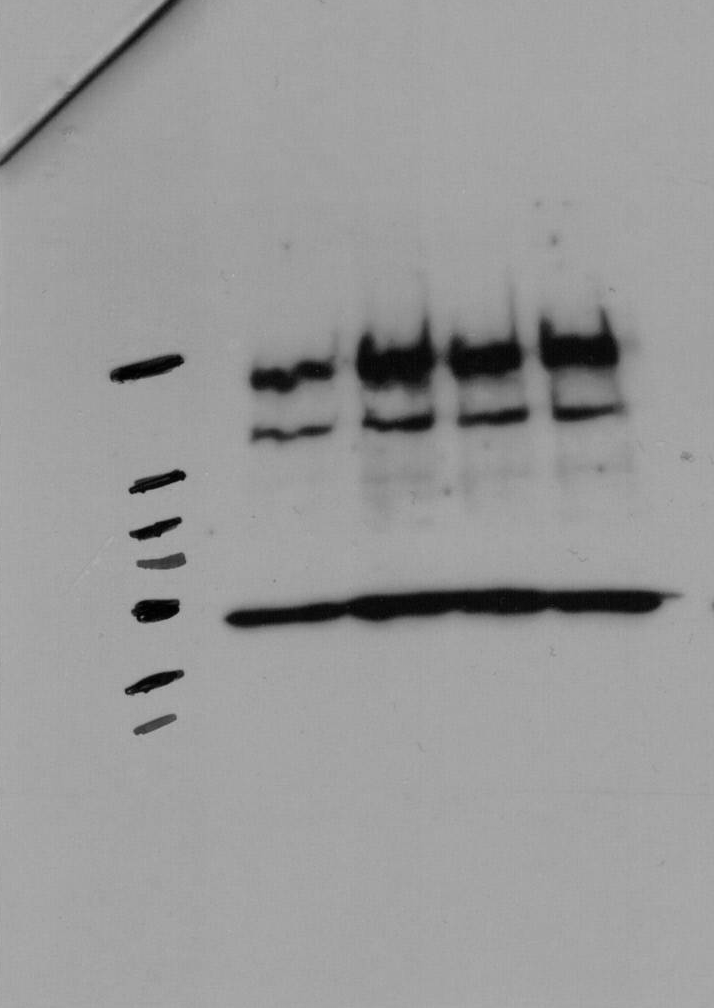

Supplement: Supplementary file 7 — Source Data [file 41467_2025_64164_MOESM7_ESM.zip › Fig. 2/Fig. 2c_MN_Munc13-1 & Act├ƒ.tif]

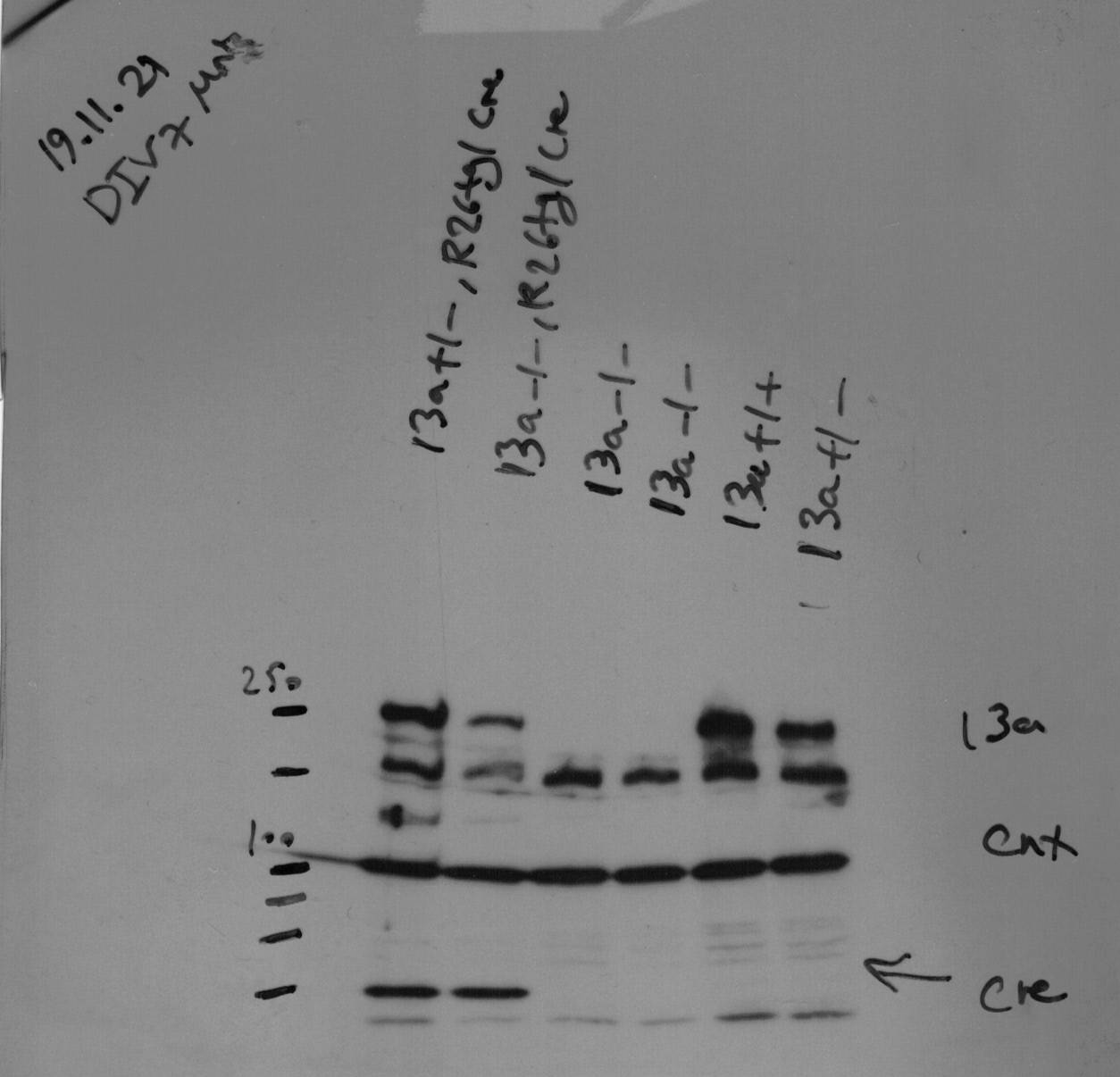

Supplement: Supplementary file 7 — Source Data [file 41467_2025_64164_MOESM7_ESM.zip › Fig. 7/Fig.7c_Munc13-1_Cre_Cnx.tif]

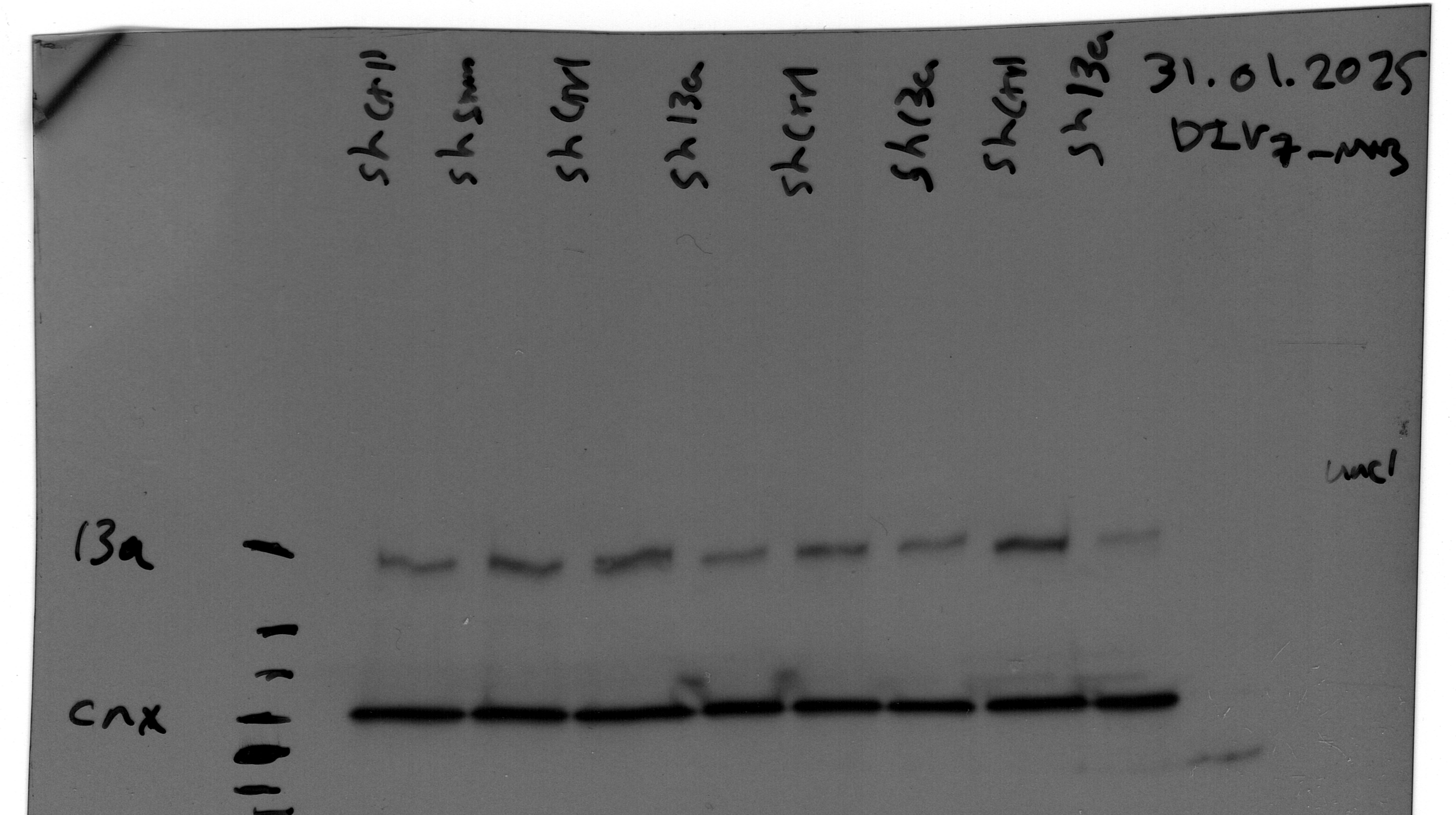

Supplement: Supplementary file 7 — Source Data [file 41467_2025_64164_MOESM7_ESM.zip › Supplementary Fig. 1/Supplementary Fig. 1b/Supplementary Fig. 1b_MN-Munc13-1 & Cnx.tif]

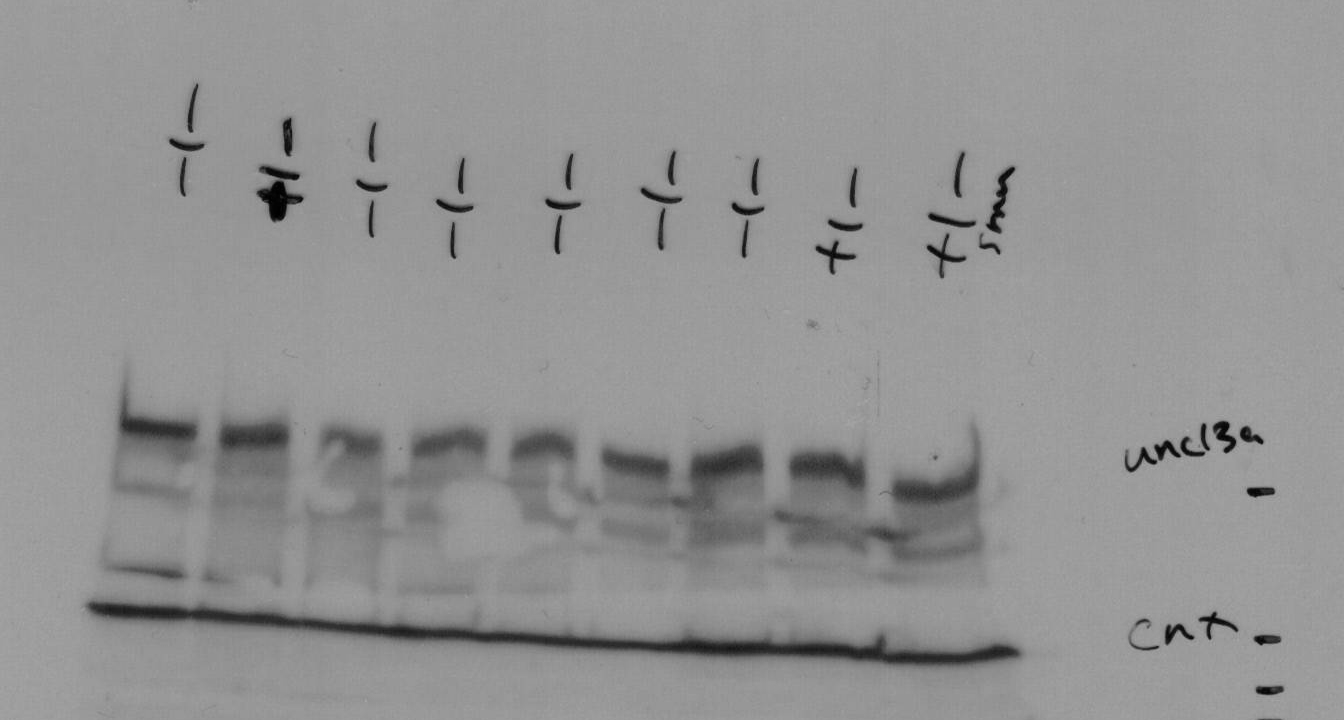

Supplement: Supplementary file 7 — Source Data [file 41467_2025_64164_MOESM7_ESM.zip › Supplementary Fig. 1/Supplementary Fig. 1i/Supplementary Fig. 1i_Munc13-1 & Cnx.tif]

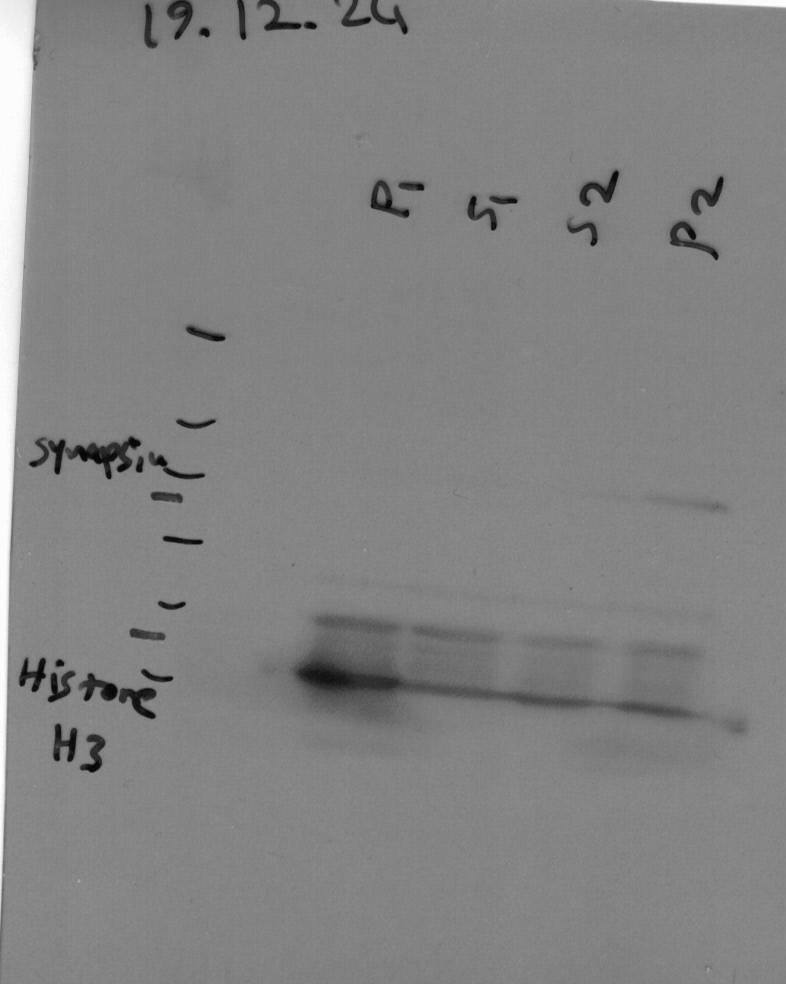

Supplement: Supplementary file 7 — Source Data [file 41467_2025_64164_MOESM7_ESM.zip › Supplementary Fig. 2/Supplementary Fig. 2f/Supplementary Fig. 2f_Histone.tif]

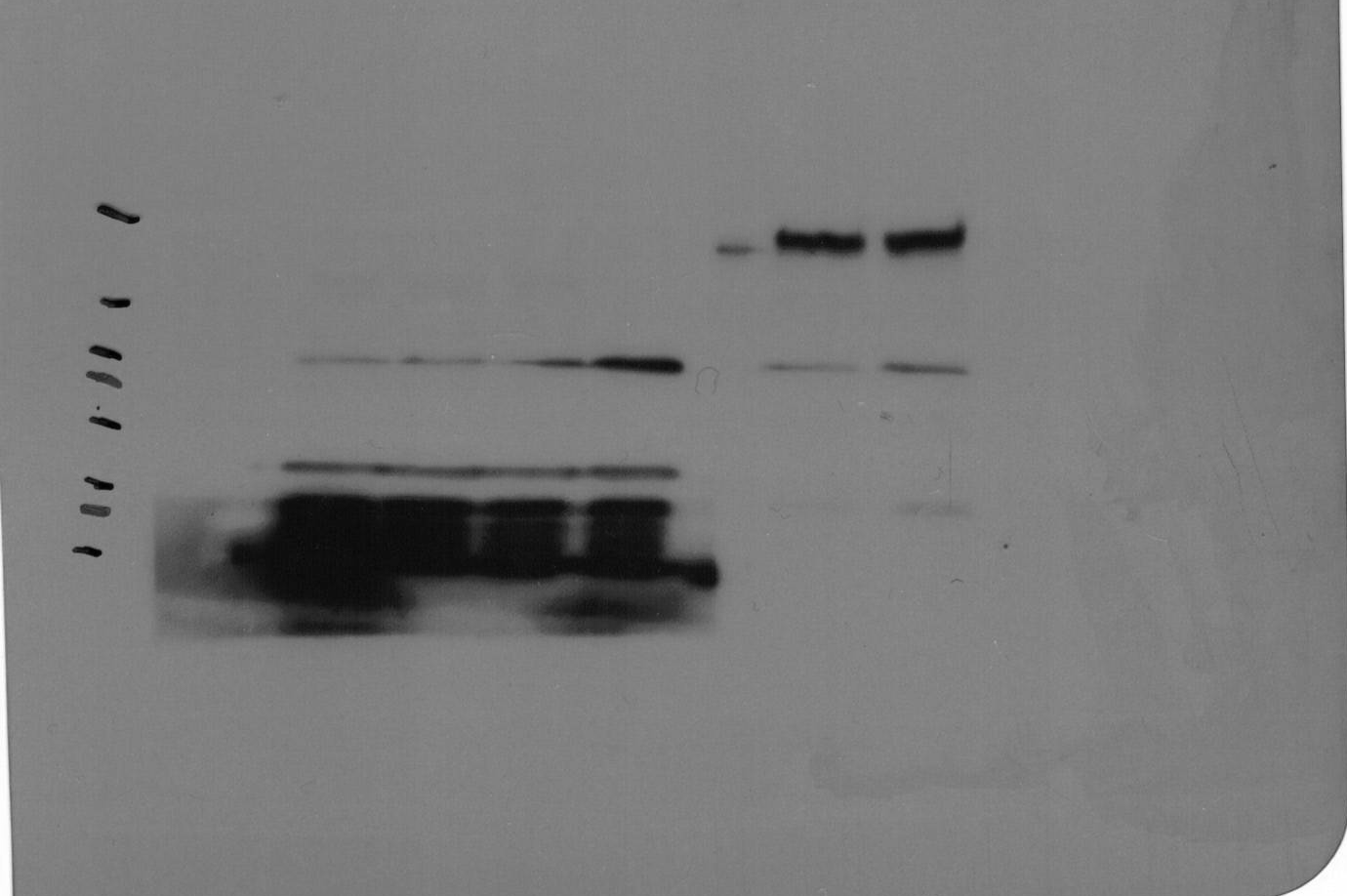

Supplement: Supplementary file 7 — Source Data [file 41467_2025_64164_MOESM7_ESM.zip › Supplementary Fig. 2/Supplementary Fig. 2f/Supplementary Fig. 2f_Synapsin.tif]

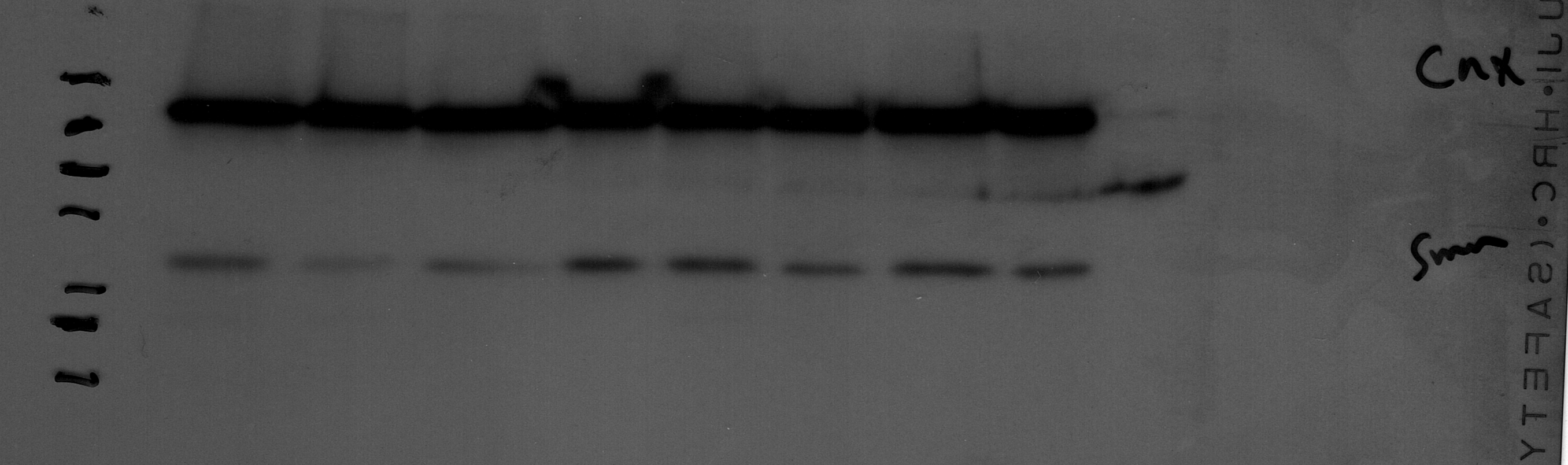

Supplement: Supplementary file 7 — Source Data [file 41467_2025_64164_MOESM7_ESM.zip › Supplementary Fig. 2/Supplementary Fig. 2g/Supplementary Fig. 2g_Smn.tif]

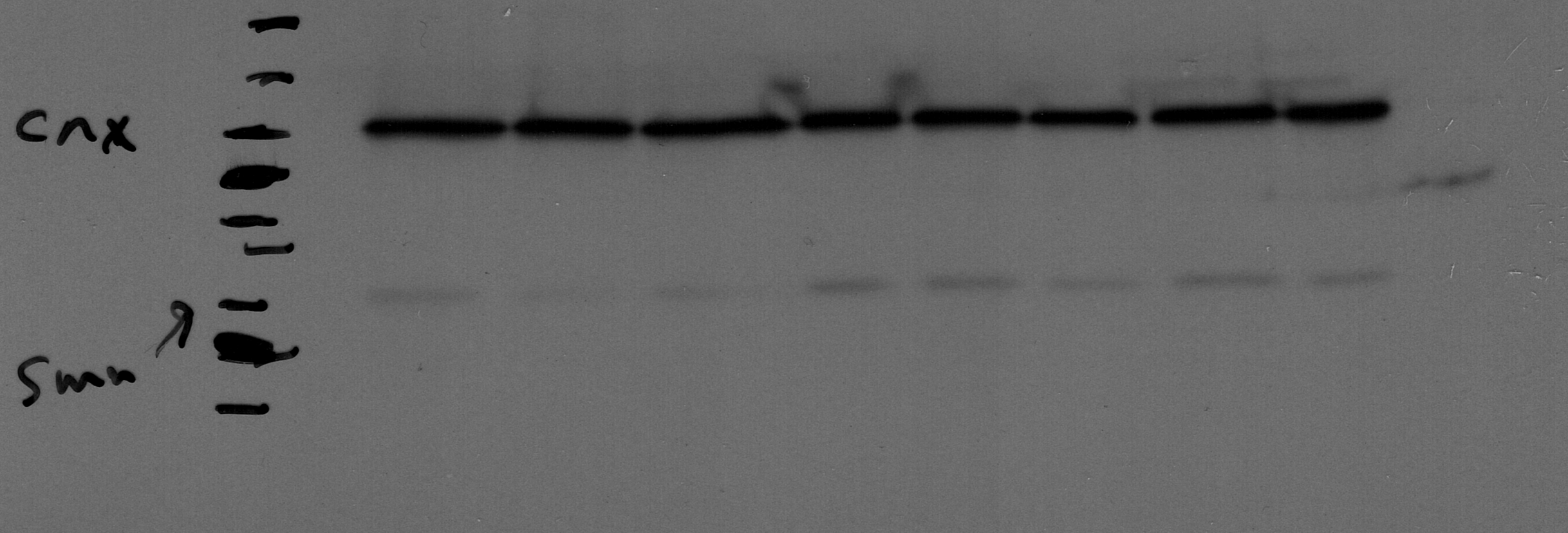

Supplement: Supplementary file 7 — Source Data [file 41467_2025_64164_MOESM7_ESM.zip › Supplementary Fig. 2/Supplementary Fig. 2g/Supplementary Fig. 2g_Cnx.tif]

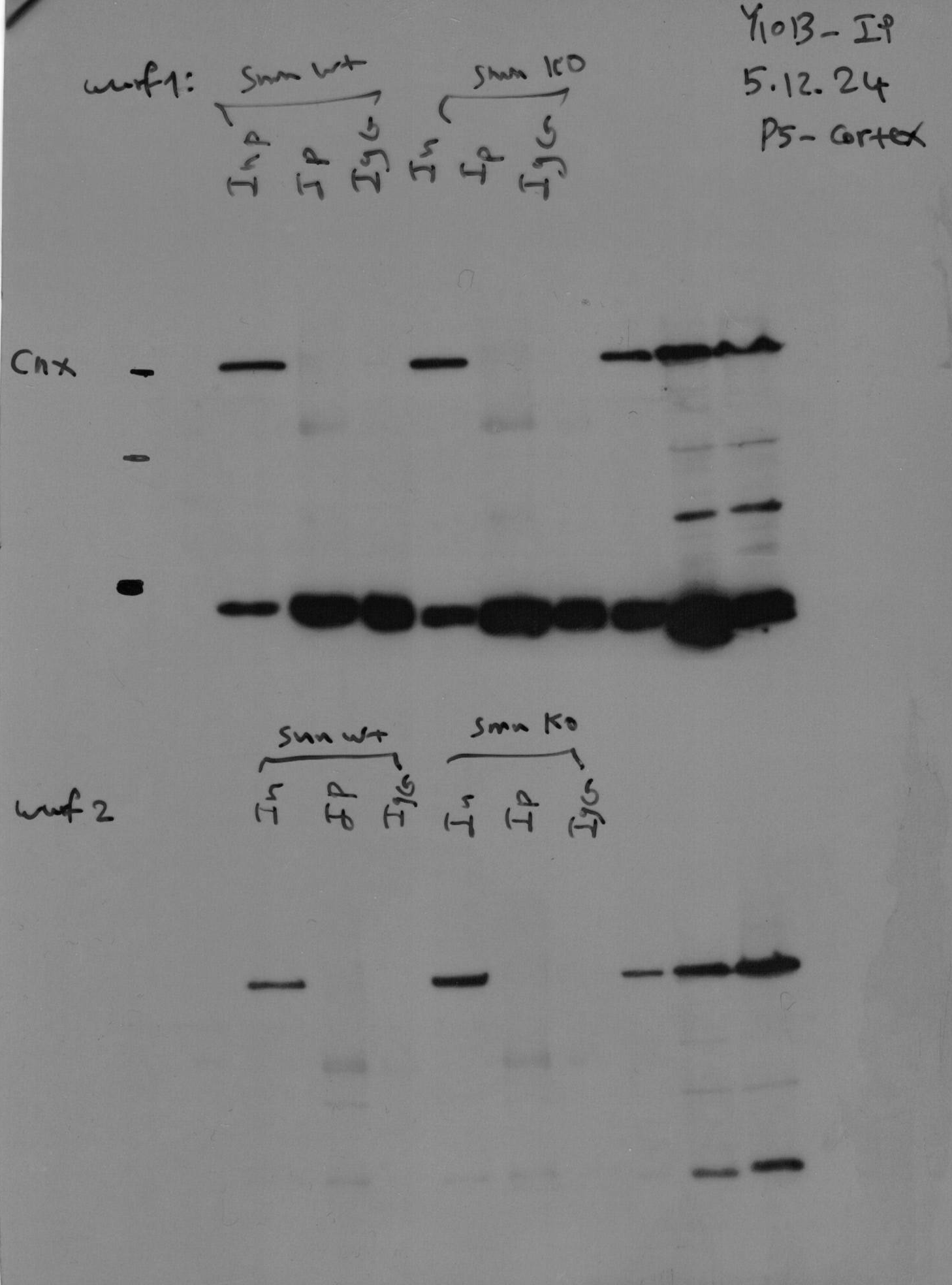

Supplement: Supplementary file 7 — Source Data [file 41467_2025_64164_MOESM7_ESM.zip › Supplementary Fig. 2/Supplementary Fig. 2a/Supplementary Fig. 2a_Cnx.tif]

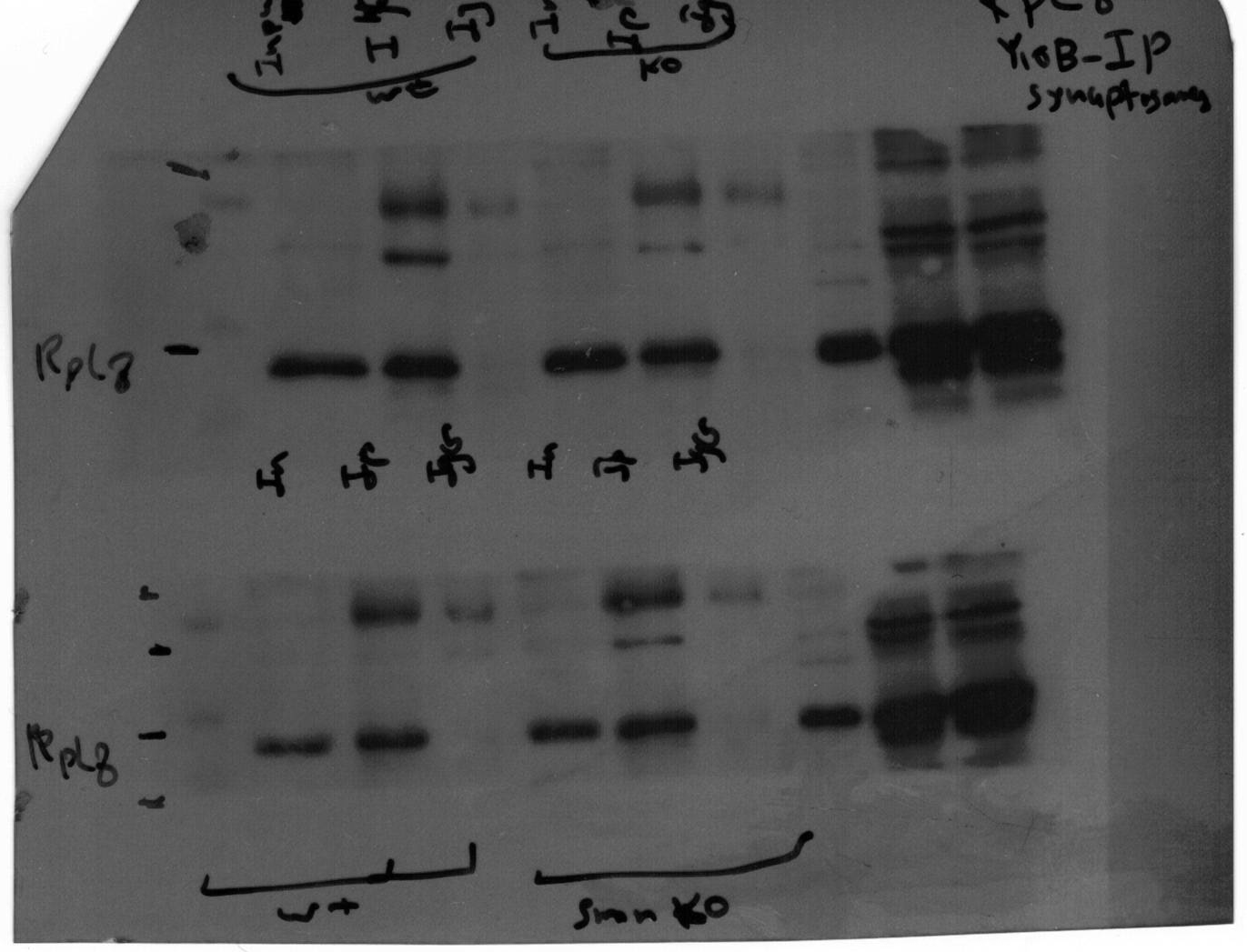

Supplement: Supplementary file 7 — Source Data [file 41467_2025_64164_MOESM7_ESM.zip › Supplementary Fig. 2/Supplementary Fig. 2a/Supplementary Fig. 2a_RPL8.tif]

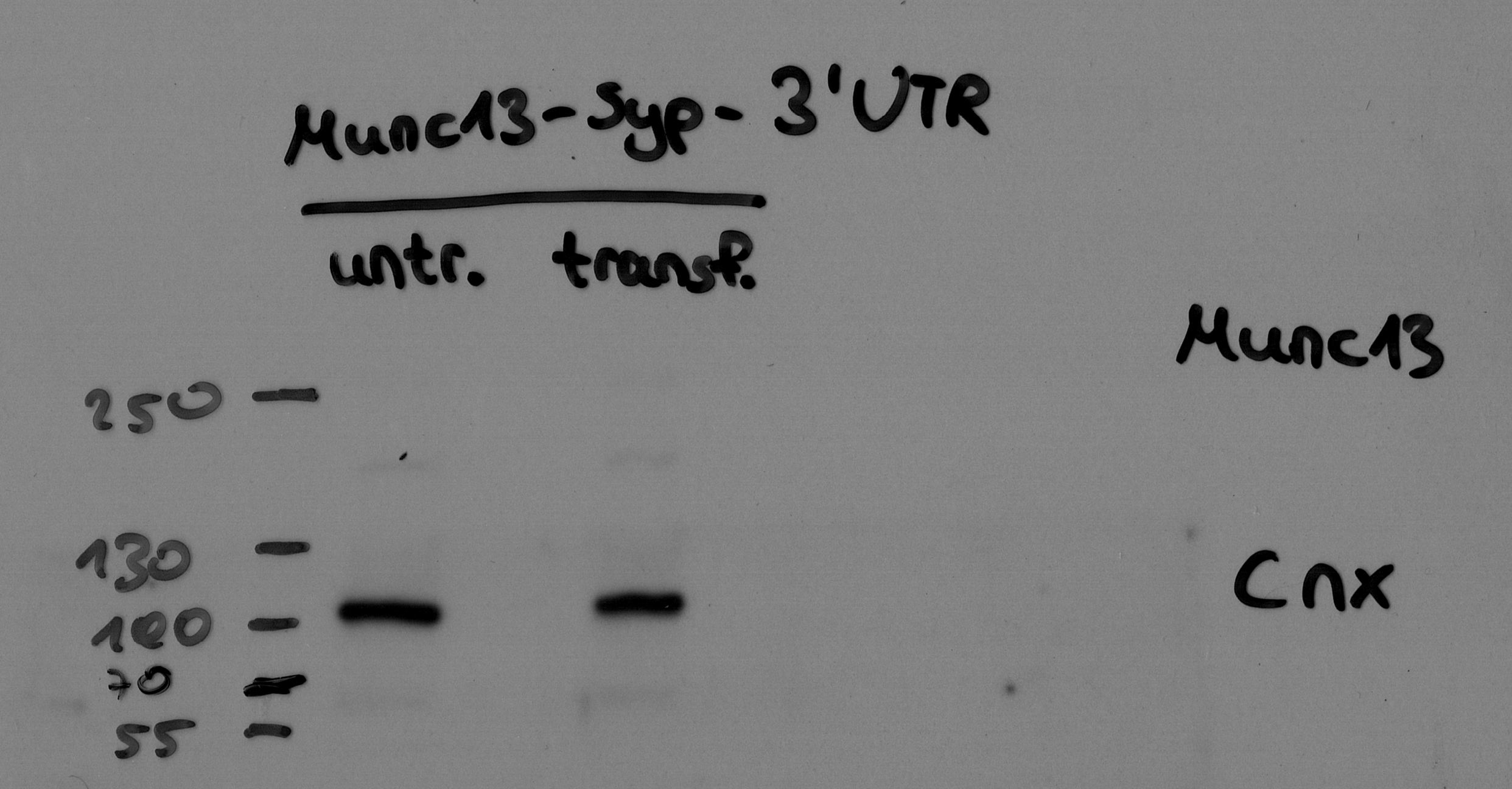

Supplement: Supplementary file 7 — Source Data [file 41467_2025_64164_MOESM7_ESM.zip › Supplementary Fig. 8/Supplementary Fig. 8d/Supplementary Fig. 8d_Cnx.tif]

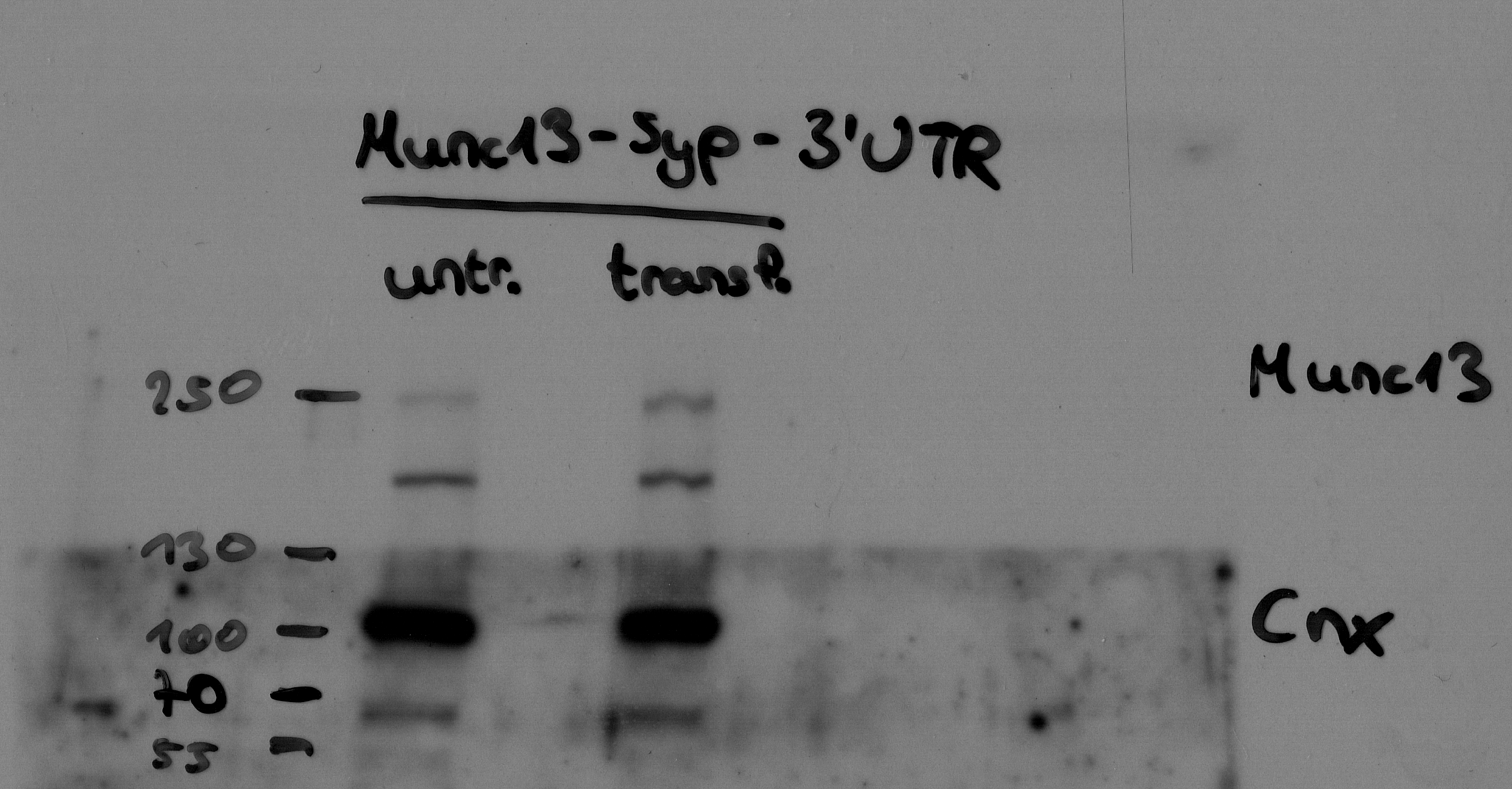

Supplement: Supplementary file 7 — Source Data [file 41467_2025_64164_MOESM7_ESM.zip › Supplementary Fig. 8/Supplementary Fig. 8d/Supplementary Fig. 8d_UNC13A.tif]

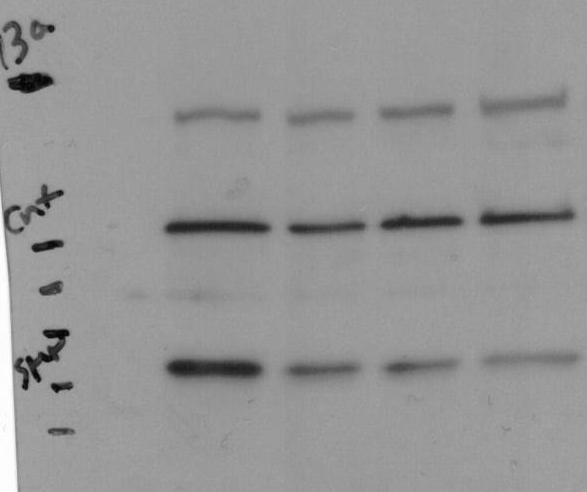

Supplement: Supplementary file 7 — Source Data [file 41467_2025_64164_MOESM7_ESM.zip › Supplementary Fig. 8/Supplementary Fig. 8b/Supplementary Fig. 8b_Cnx & SMN.tif]
